# Suicide among physicians and health-care workers: A systematic review and meta-analysis

**DOI:** 10.1371/journal.pone.0226361

**Published:** 2019-12-12

**Authors:** Frédéric Dutheil, Claire Aubert, Bruno Pereira, Michael Dambrun, Fares Moustafa, Martial Mermillod, Julien S. Baker, Marion Trousselard, François-Xavier Lesage, Valentin Navel

**Affiliations:** 1 Université Clermont Auvergne, CNRS, LaPSCo, Physiological and Psychosocial Stress, CHU Clermont-Ferrand, University Hospital of Clermont-Ferrand, Occupational and Preventive Medicine, WittyFit, Clermont-Ferrand, France; 2 Australian Catholic University, Faculty of Health, School of Exercise Science, Melbourne, Victoria, Australia; 3 Université de Versailles Saint-Quentin-en-Yvelines, Faculty of Health Science Simone Veil, Versailles, France; 4 CHU Clermont-Ferrand, University Hospital of Clermont-Ferrand, Biostatistics Unit, the Clinical Research and Innovation Direction, Clermont-Ferrand, France; 5 Université Clermont Auvergne, CNRS, LaPSCo, Physiological and Psychosocial Stress, Clermont-Ferrand, France; 6 CHU Clermont-Ferrand, University Hospital of Clermont-Ferrand, Emergency, Clermont-Ferrand, France; 7 Univ. Grenoble Alpes, Univ. Savoie Mont Blanc, CNRS, LPNC, Grenoble, France; 8 Institut Universitaire de France, Paris, France; 9 Centre for Health and Exercise Science Research, Department of Sport, Physical Education and Health, Hong Kong Baptist University, Kowloon Tong, Hong Kong; 10 French Armed Forces Biomedical Research Institute-IRBA, Neurophysiology of Stress, Neuroscience and Operational Constraint Department, Brétigny-sur-Orge, France; 11 University of Montpellier, Laboratory Epsylon EA, Dynamic of Human Abilities & Health Behaviors, CHU Montpellier, University Hospital of Montpellier, Occupational and Preventive Medicine, Montpellier, France; 12 CHU Clermont-Ferrand, University Hospital of Clermont-Ferrand, Ophthalmology, Clermont-Ferrand, France; Yokohama City University, JAPAN

## Abstract

**Background:**

Medical-related professions are at high suicide risk. However, data are contradictory and comparisons were not made between gender, occupation and specialties, epochs of times. Thus, we conducted a systematic review and meta-analysis on suicide risk among health-care workers.

**Method:**

The PubMed, Cochrane Library, Science Direct and Embase databases were searched without language restriction on April 2019, with the following keywords: suicide* AND (« health care worker* » OR physician* OR nurse*). When possible, we stratified results by gender, countries, time, and specialties. Estimates were pooled using random-effect meta-analysis. Differences by study-level characteristics were estimated using stratified meta-analysis and meta-regression. Suicides, suicidal attempts, and suicidal ideation were retrieved from national or local specific registers or case records. In addition, suicide attempts and suicidal ideation were also retrieved from questionnaires (paper or internet).

**Results:**

The overall SMR for suicide in physicians was 1.44 (95CI 1.16, 1.72) with an important heterogeneity (I^2^ = 93.9%, p<0.001). Female were at higher risk (SMR = 1.9; 95CI 1.49, 2.58; and ES = 0.67; 95CI 0.19, 1.14; p<0.001 compared to male). US physicians were at higher risk (ES = 1.34; 95CI 1.28, 1.55; p <0.001 vs Rest of the world). Suicide decreased over time, especially in Europe (ES = -0.18; 95CI -0.37, -0.01; p = 0.044). Some specialties might be at higher risk such as anesthesiologists, psychiatrists, general practitioners and general surgeons. There were 1.0% (95CI 1.0, 2.0; p<0.001) of suicide attempts and 17% (95CI 12, 21; p<0.001) of suicidal ideation in physicians. Insufficient data precluded meta-analysis on other health-care workers.

**Conclusion:**

Physicians are an at-risk profession of suicide, with women particularly at risk. The rate of suicide in physicians decreased over time, especially in Europe. The high prevalence of physicians who committed suicide attempt as well as those with suicidal ideation should benefits for preventive strategies at the workplace. Finally, the lack of data on other health-care workers suggest to implement studies investigating those occupations.

## Introduction

Suicide risk was increased in certain occupational groups, especially in medical-related professions [1]. Physicians, and other health-care workers such as nurses [2,3], were considered like high risk group of suicide in different countries [4,5,6], especially for women [6,7,8]. Indeed, despite considerably higher risk of suicides in men than women in the general population [9], female doctors have higher suicide rates than men [10], putatively because of their social family role [11], or a poor status integration within the profession [7]. Suicide rate in physicians was also not homogenous in all countries [12], and physicians’ satisfaction has been reported to change between different epochs of times [13]. Physicians working conditions varied substantially between countries and over contemporary times, these factors were never investigated in relationships with suicide in physicians. For example, there were tentative to regulate working time of physicians over the recent years, such as in Europe with its European Working Time Directive (EWTD) [14]. Some specialties have been suggested to be particularly at risk of suicides [15,16] with occupational factors individualized in different medical or surgical specialties: heavy workload and working hours involved in the job such as long shifts and unpredictable hours (with the sleep deprivation associated) [17], stress of the situations (life and death emergencies) [18], and easy access to a means of committing suicide [19]. To implement coordinated and synergistic preventive strategies, we need to identify physicians in mental health suffering [20], therefore statistical analyses on suicide attempts and suicidal ideation were necessary. However, robust statistics on health-care workers were desperately lacking for suicides, suicide attempts and suicidal ideation. The latest meta-analysis summarized physicians suicide risk before 2000s [6], we need for updated synthesis of literature. We hypothesized that 1) physicians are more at risk to commit suicide than the general population, 2) women physicians are more at risk to commit suicide than their male counterparts, 3) some countries would have higher rates of suicide in physicians, 4) with an improvement over time, 5) some medical or surgical specialties would be at higher risk of suicide, 6) physicians would also exhibit higher rates of suicide attempts and suicidal ideation, and 7) other health care workers would also be at risk of suicide.

Thus, we aimed to conduct a systematic review of the literature and meta-analysis to provide evidence-based data for suicide risk among health-care workers, considering gender, geographic zone, epoch of time, medical and surgical specialties. Finally, we wanted to expand our study to suicide attempts and suicidal ideation.

## Methods

### Search strategy and study eligibility

**Fig 1:**
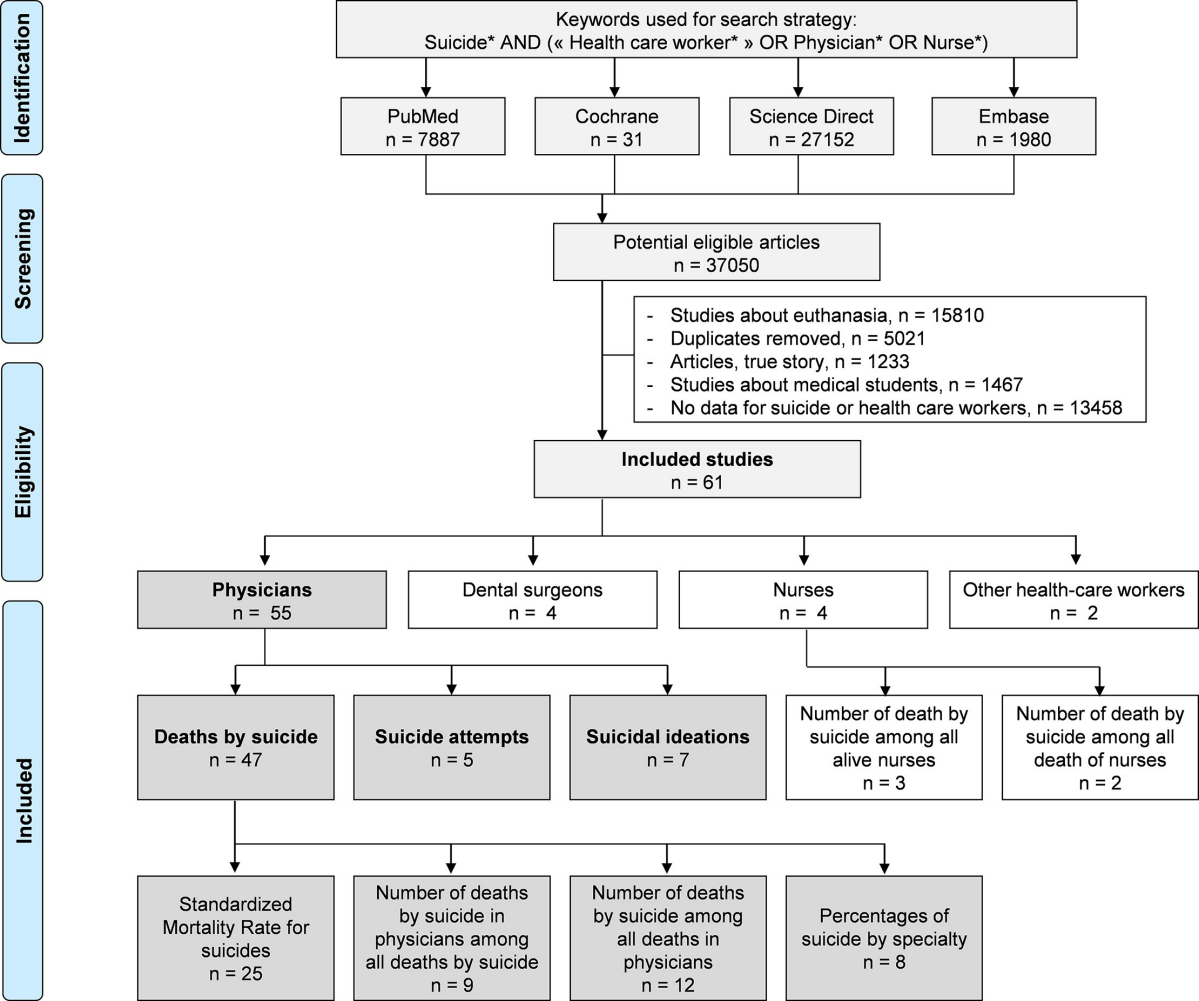
Search strategy.

We reviewed all studies involving suicides, suicide attempts or suicidal ideation in health-care workers. Students were excluded because of the difference in responsibilities in comparisons with health-care workers, and because of the existence of previous recent meta-analyses focusing specifically on health-care students [21,22,23,24]; we included interns because they were not included in the aforementioned meta-analyses on prevalence of suicides, suicide attempts or suicidal ideation, and because they could have similar responsibilities to senior practitioners. The PubMed, Cochrane Library, Science Direct and Embase databases were searched on April 2019, with the following keywords: suicide* AND (« health care worker* » OR physician* OR nurse*). The search was not limited by years or languages. To be included, articles had to be peer-reviewed and to describe original empirical data on suicides, suicide attempt or suicidal ideation in health-care workers. When data were available, we also collected data from a control group (such as general population) for comparisons purposes. In addition, reference lists of all publications meeting the inclusion criteria will be manually searched to identify any further studies not found through digital research. The search strategy was presented in Fig 1. Three authors (Claire Aubert, Valentin Navel and Frederic Dutheil) conducted all literature searches, and separately reviewed the abstracts and decided the suitability of the articles for inclusion. Two others authors (Bruno Pereira and Martial Mermillod) have been asked to review the articles when consensus on suitability was debated. Then all authors reviewed the eligible articles.

### Quality of assessment

**Fig 2:**
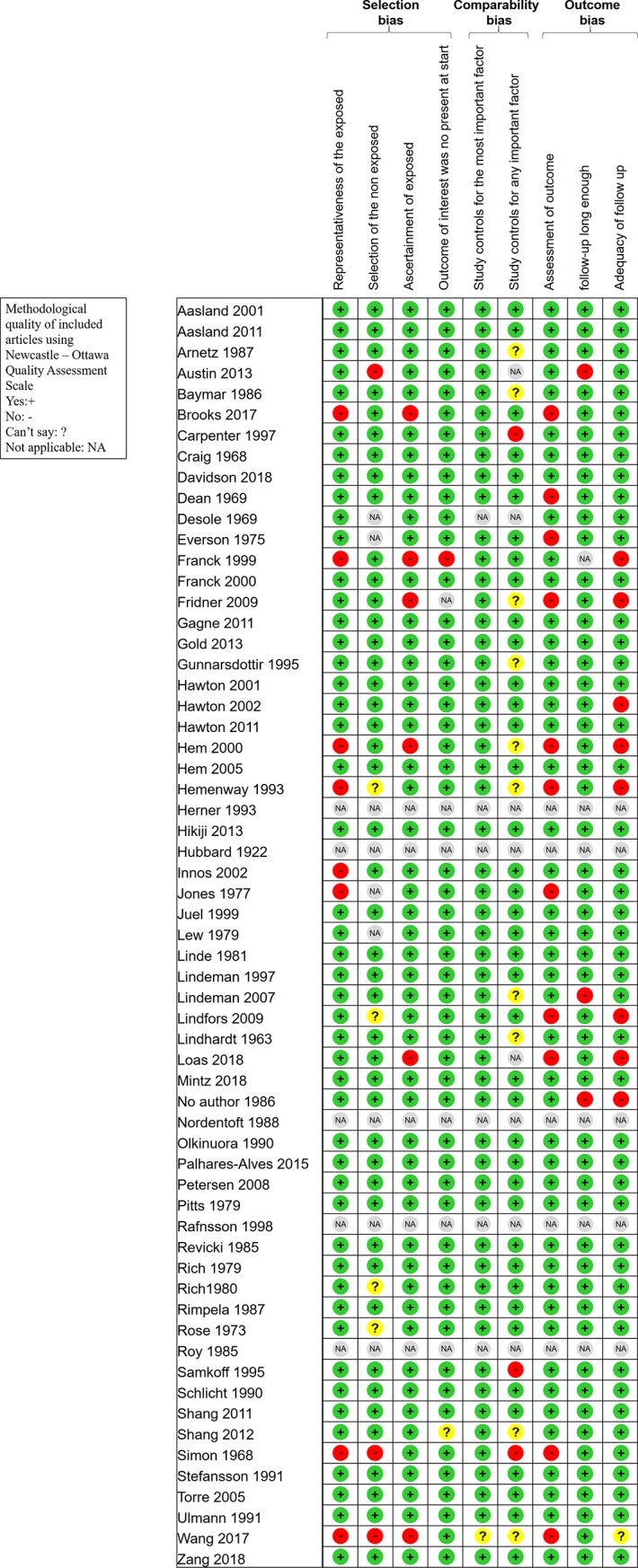
Methodological quality of included articles using Newcastle–Ottawa Quality Assessment Scale.

**Fig 3:**
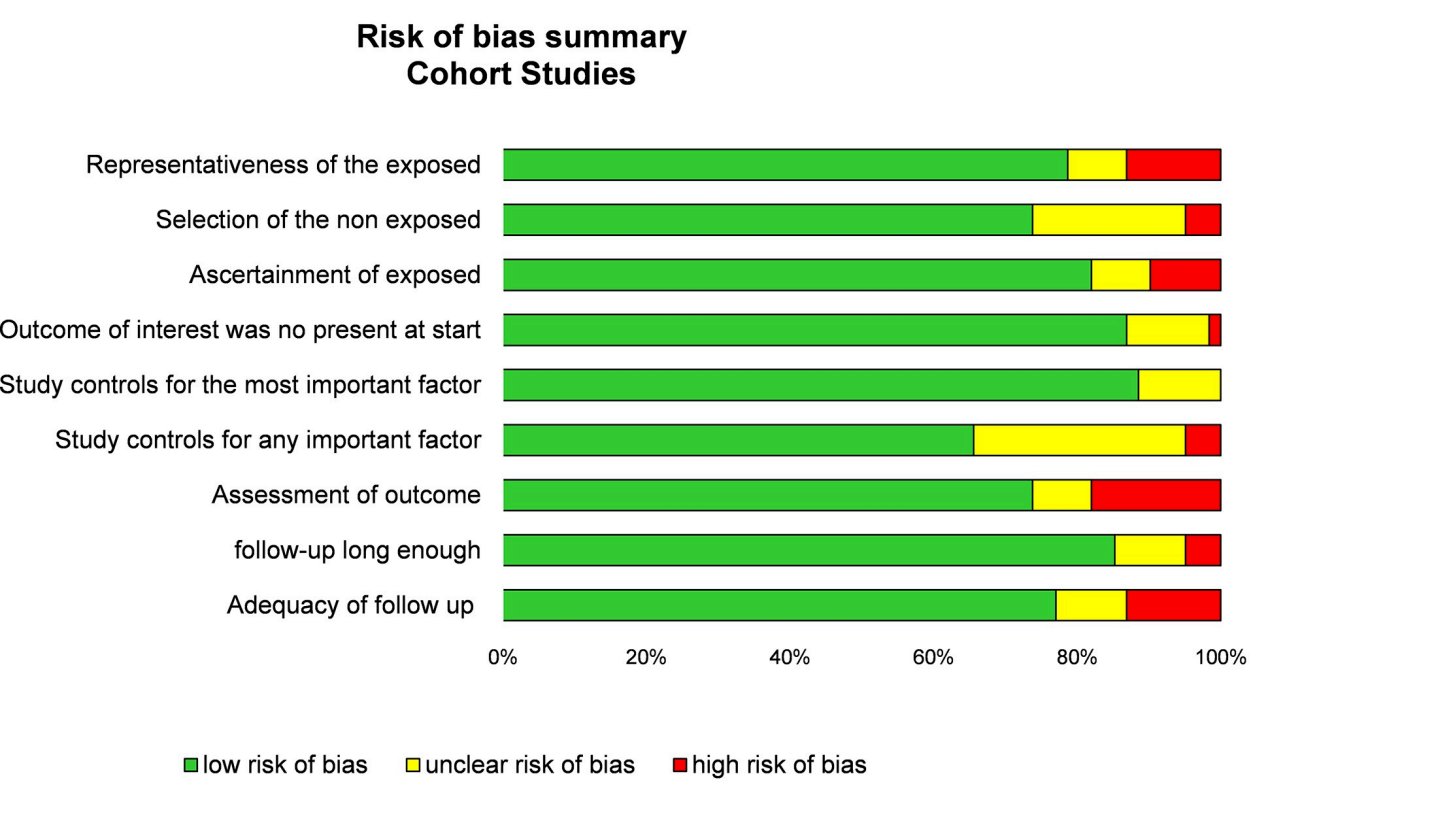
Summary bias risk of included articles using the Newcastle–Ottawa Quality Assessment Scale model.

Although not designed for quantifying the integrity of studies [25], the “STrengthening the Reporting of Observational studies in Epidemiology” (STROBE) criteria [26] and Newcastle-Ottawa Scale (NOS) were used to check the quality of articles [27]. The maximum score in STROBE criteria was 30 with assessment of 22 items, in NOS criteria was 9 with assessment of 8 items (one star for each item within the selection and exposure category and a maximum of two stars for comparability) (Figs 2 and 3).

## Statistical considerations

**Fig 4:**
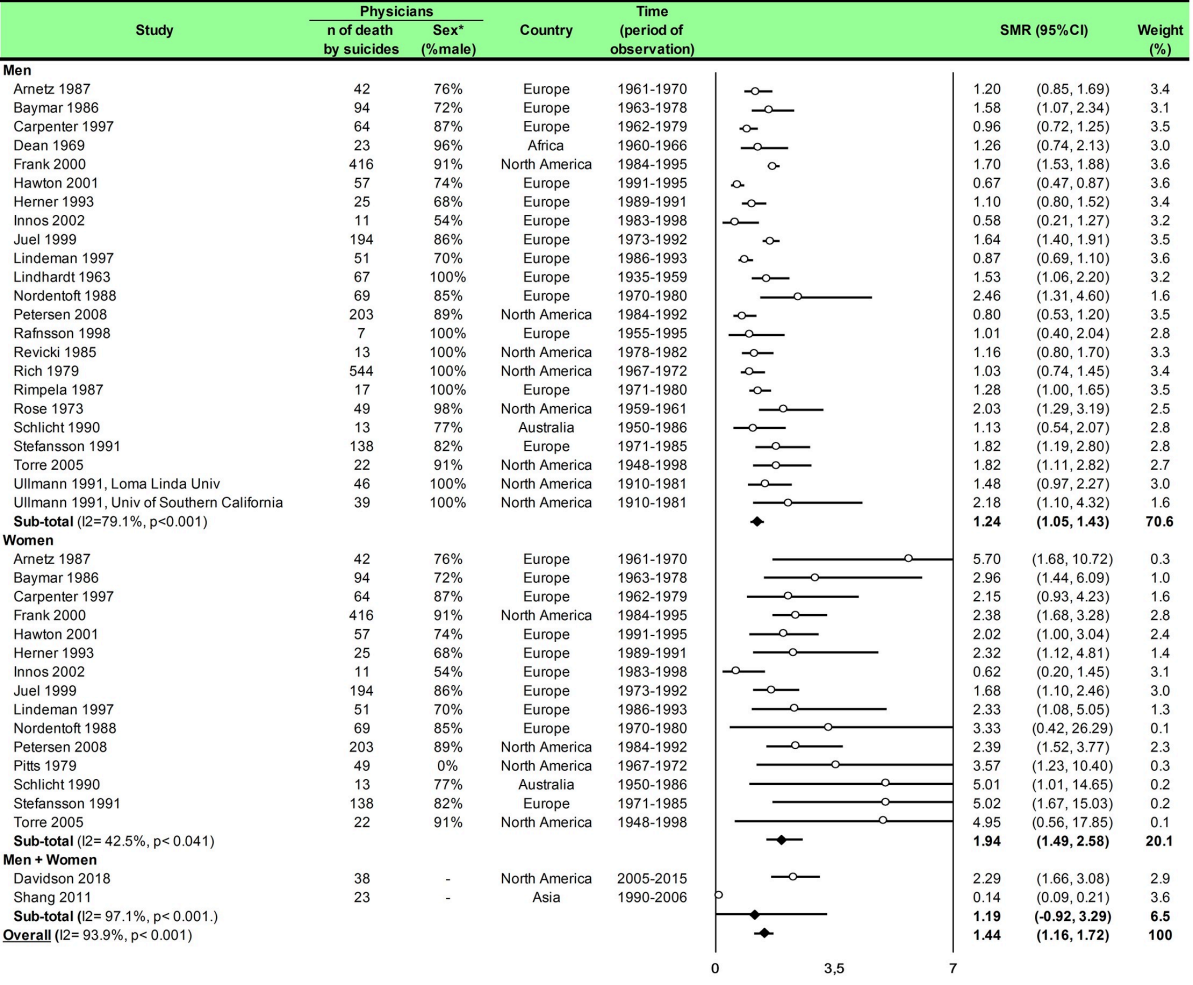
Meta-analysis of standardized mortality rate for suicides among physicians by gender.

**Fig 5:**
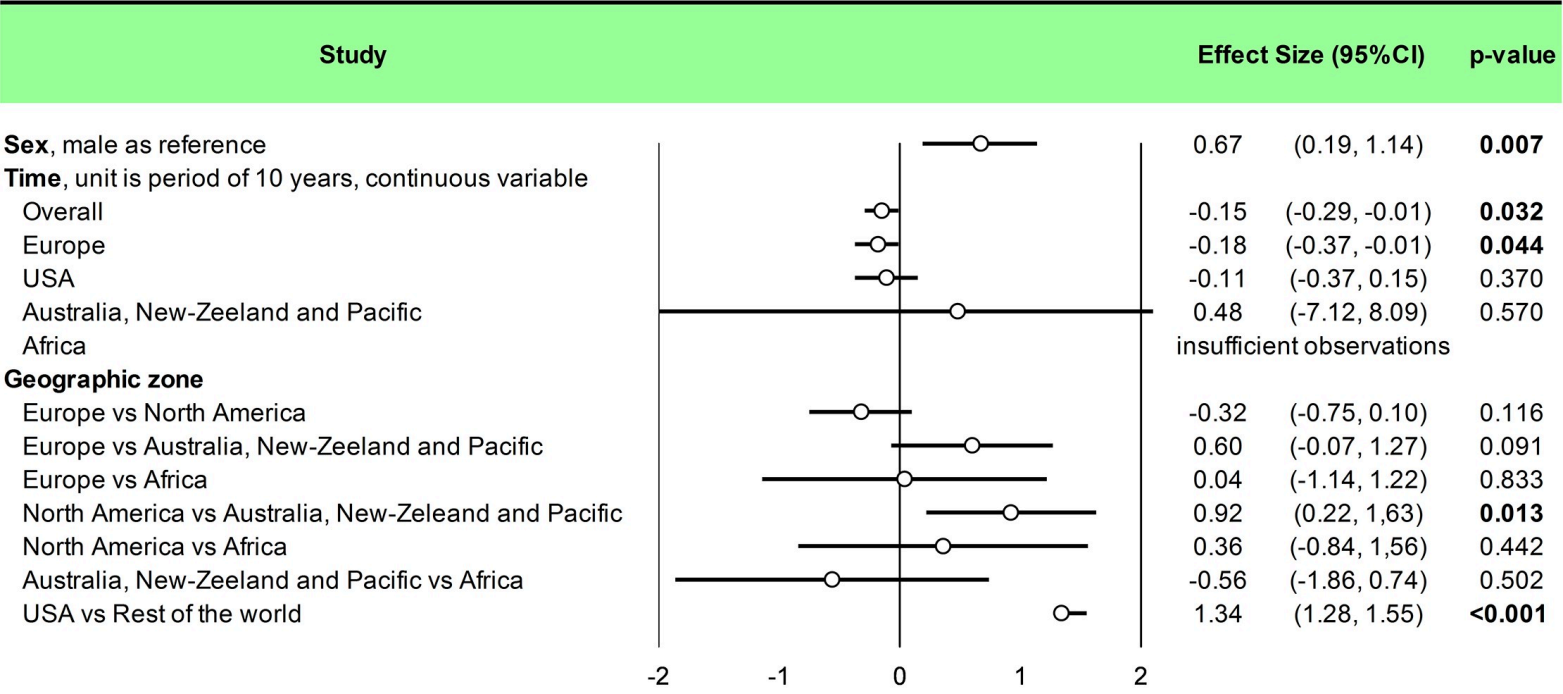
Meta-regression of standardized mortality rate for suicides among physicians.

**Fig 6:**
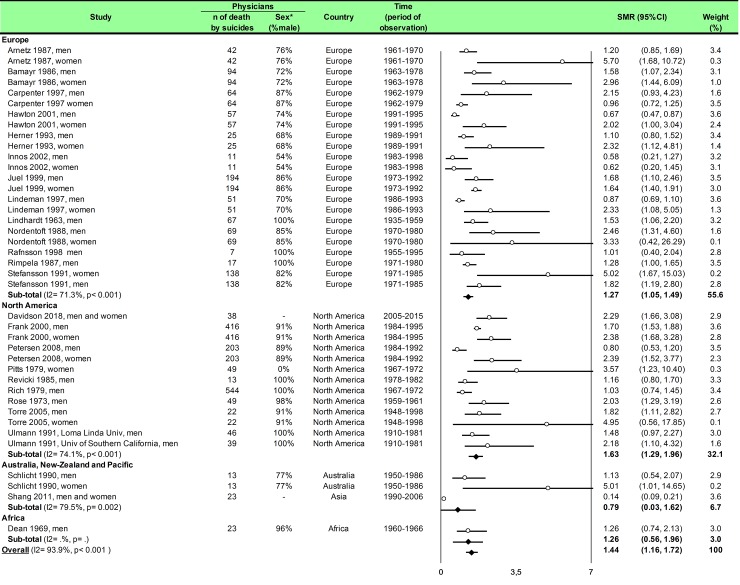
Meta-analysis of standardized mortality rate for suicides by geographic zones.

**Fig 7:**
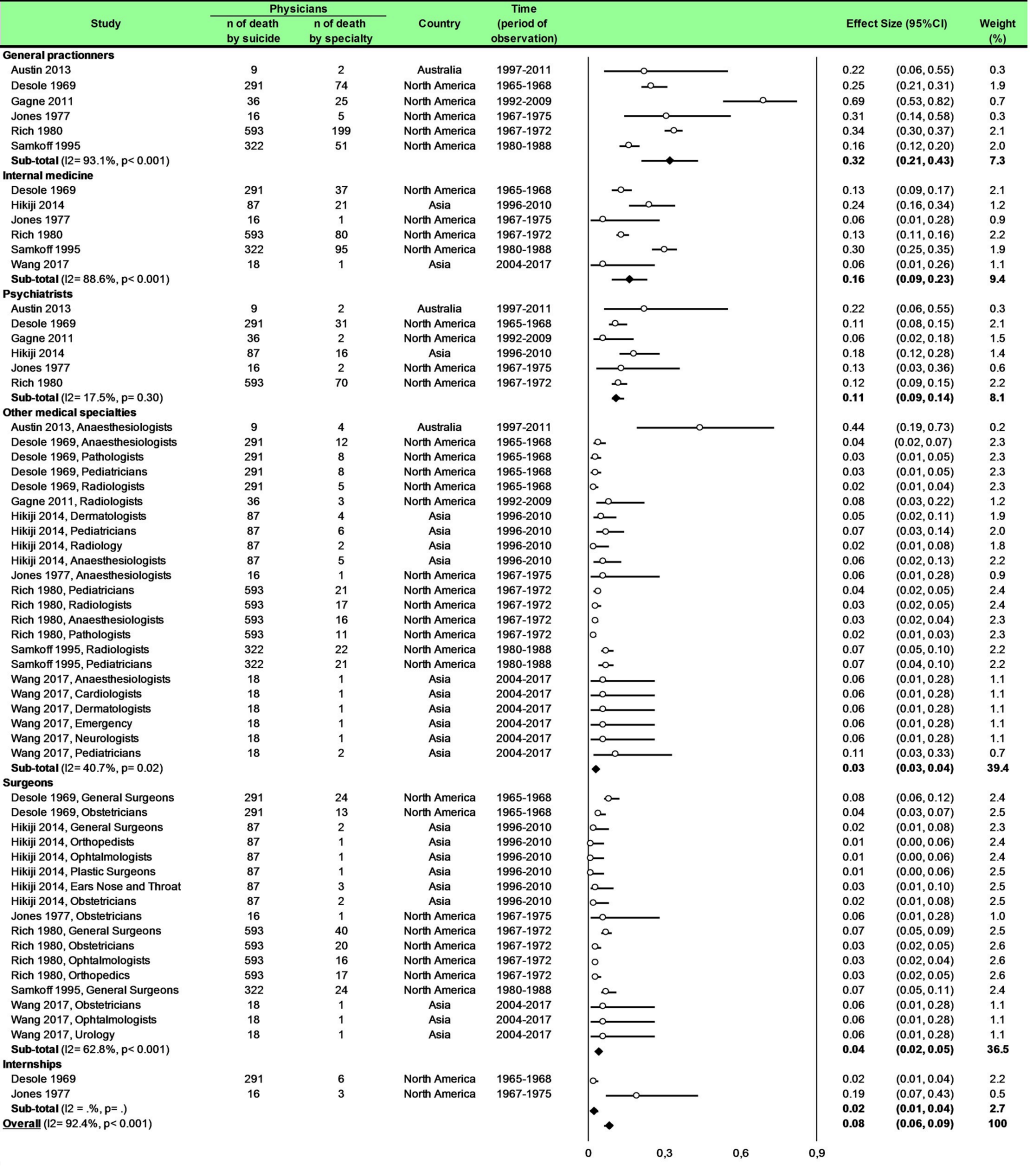
Meta-analysis of percentages of suicide in physicians by group of specialties.

**Fig 8:**
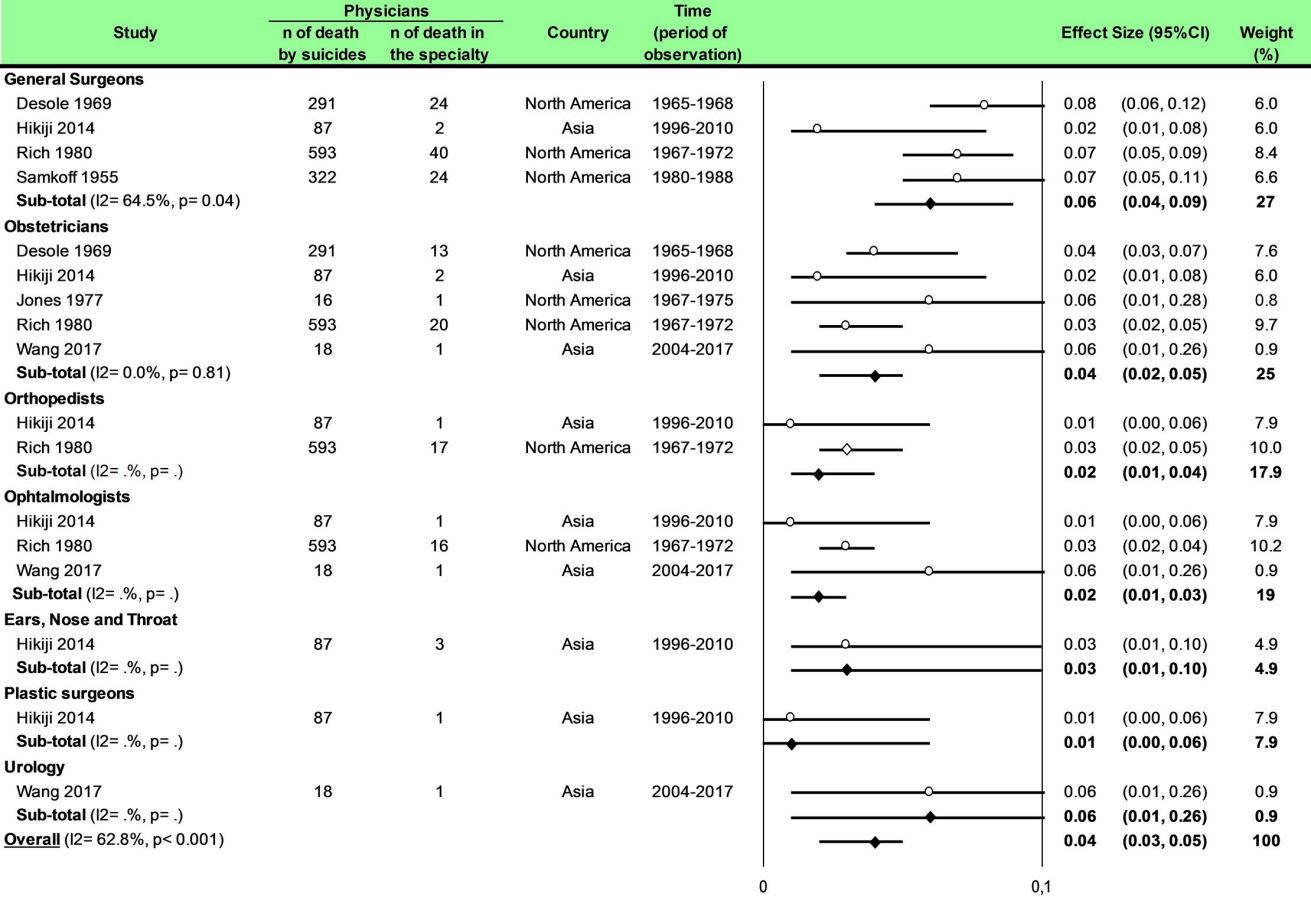
Meta-analysis of percentages of suicide in physicians by category of surgical specialties.

**Fig 9:**
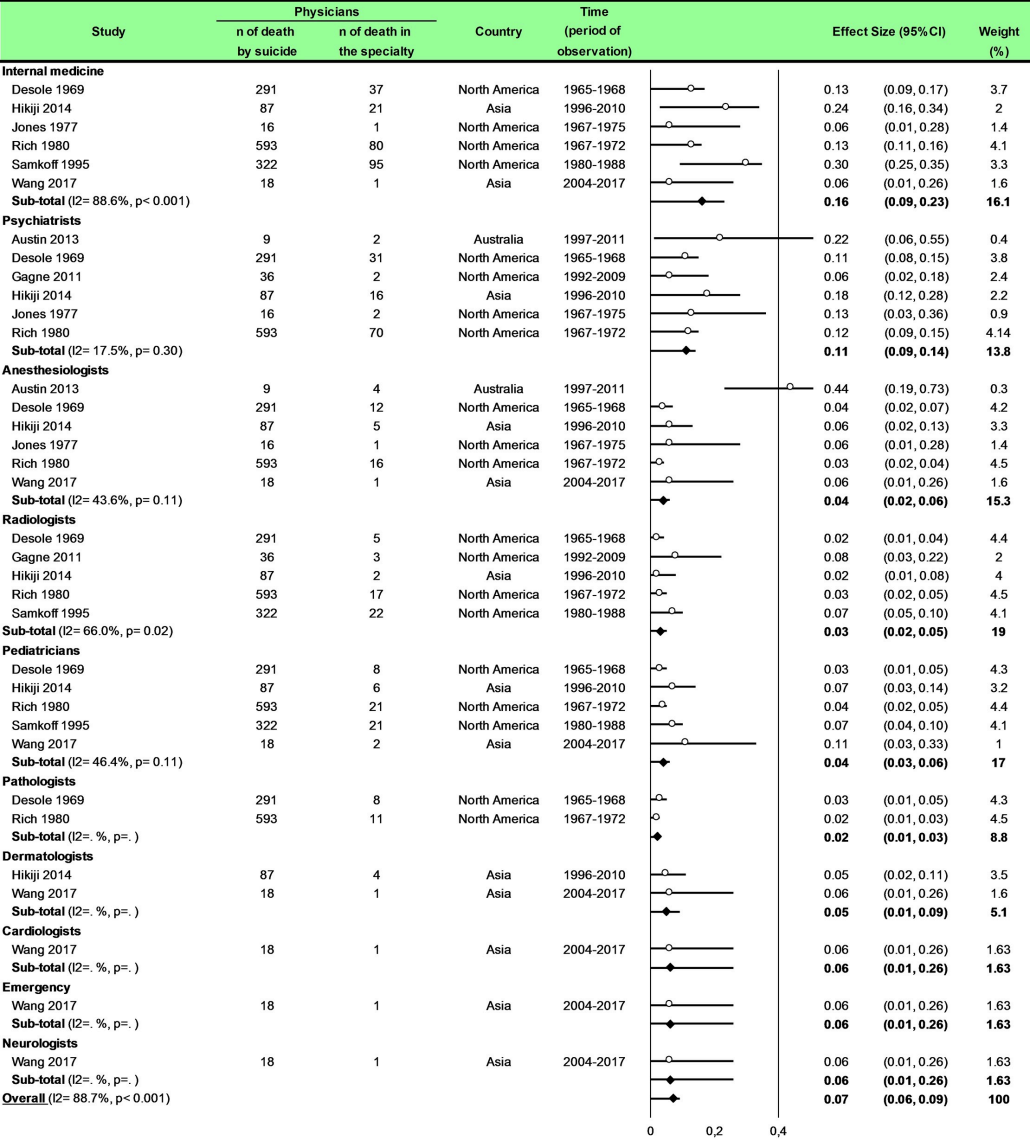
Meta-analysis of percentages of suicide in physicians by category of medical specialties.

**Fig 10:**
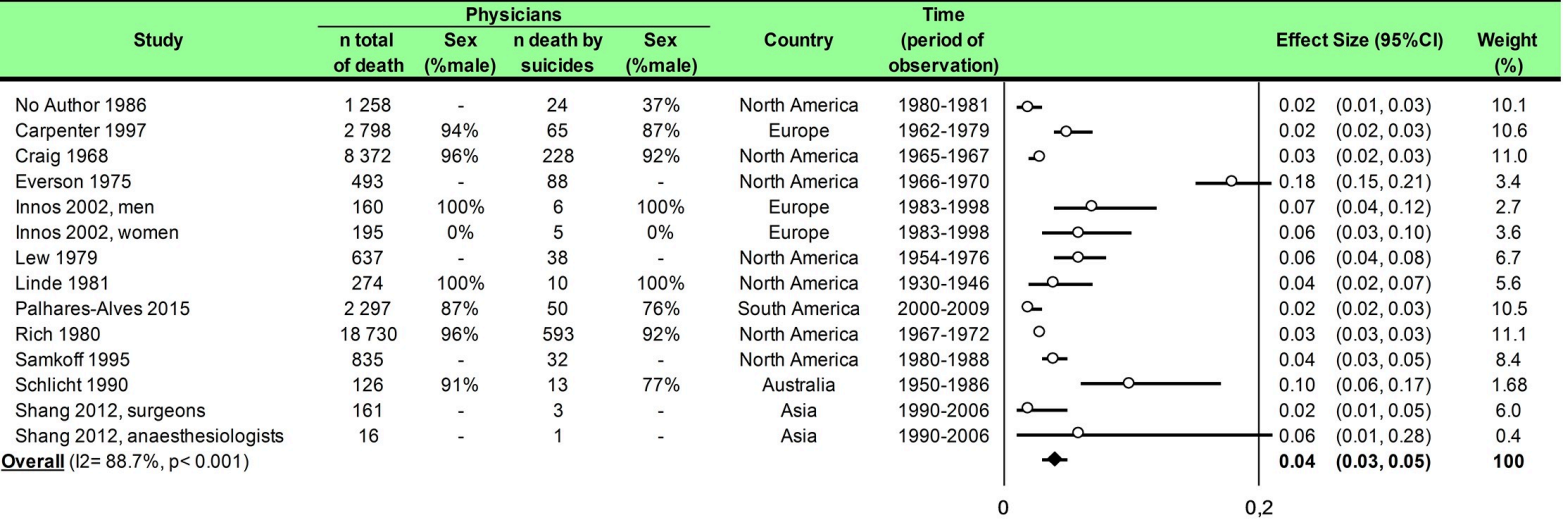
Meta-analysis of prevalence of physicians died by suicide among all deaths in physicians.

**Fig 11:**
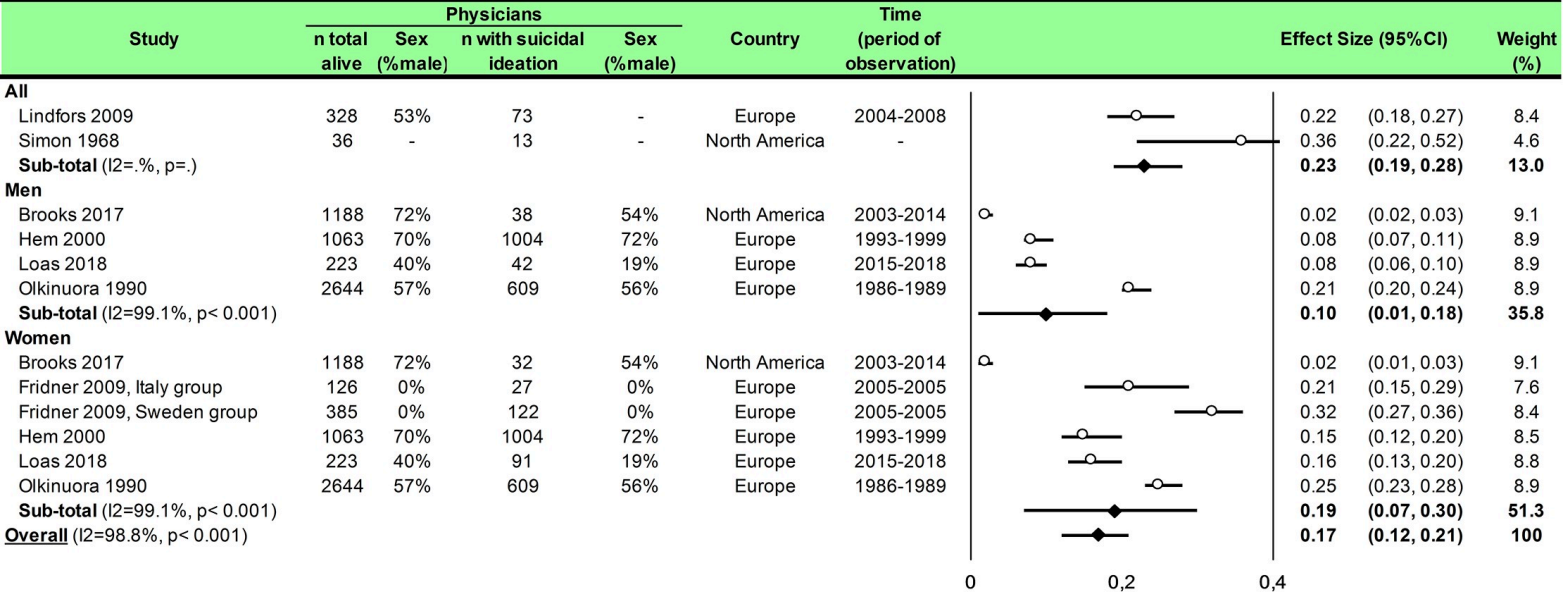
Meta-analysis of prevalence of physicians with suicidal ideation among all the physicians.

Statistical analysis was conducted using Comprehensive Meta-analysis software (version 2, Biostat Corporation) [28,29,30] and Stata software (version 13, StataCorp, College Station, US) [28,29,31]. Main characteristics were summarized for each study sample and reported as mean (standard-deviation) and number (%) for continuous and categorical variables respectively. Statistical heterogeneity between results was assessed by examining forest plots, confidence intervals (CI) and using formal tests for homogeneity based on I^2^ statistic, which is the most common metric for measuring the magnitude of heterogeneity between studies and is easily interpretable. I^2^ values range between 0% and 100% and are typically considered low for <25%, moderate for 25–50%, and high for > 50%. Random effect meta-analysis (DerSimonian and Liard approach) were conducted when data could be pooled [32]. P values < 0.05 were considered statistically significant. We conducted: 1) meta-analyses on the Standardized Mortality Ratio (SMR) for suicides i.e. the ratio between the observed and expected number of death among physicians, stratified by sex (Fig 4; and Fig 5 for metaregressions), geographic zones (Fig 6), epochs of time, and by categories of specialties (main groups of specialities (Fig 7 and S1 Fig), surgical specialties (Fig 8 and S2 Fig), then medical specialities (Fig 9 and S3 Fig), 2) meta-analyses on the prevalence of health-care workers died by suicide among all health-care workers death (Fig 10), 3) meta-analyses on the prevalence of health-care workers died by suicide among all the deaths by suicide in the general population (S4 Fig), 4) meta-analyses on suicide attempts (S5 Fig) and suicidal ideation (Fig 11). Effect-size was estimated for quantitative endpoints as number of physicians having done suicide attempt and number of physicians with suicidal ideation. A scale for ES has been suggested with 0.8 reflecting a large effect, 0.5 a moderate effect, and 0.2 a small effect [33]. When possible (sufficient sample size), meta-regressions were proposed to study relation between prevalence and epidemiological relevant parameters determined according to the literature: sex, geographic zone, epoch of time (for studies with a follow-up over several consecutive years, we based our statistics on the mean year of epoch of time). Results were expressed as regression coefficient and 95% CI.

## Results

An initial search produced a possible 37050 articles (Fig 1). Removal of duplicates and use of the selection criteria reduced the search to 61 articles [1,2,5,7,8,15,16,34,35,36,37,38,39,40,41,42,43,44,45,46,47,48,49,50,51,52,53,54,55,56,57,58,59,60,61,62,63,64,65,66,67,68,69,70,71,72,73,74,75,76,77,78,79,80,81,82,83,84,85,86,87]. In those 61 articles, 55 articles were on physicians [1,5,7,8,15,16,34,35,36,37,38,39,40,41,42,43,44,45,46,47,48,49,50,51,52,53,54,55,56,57,58,59,60,61,62,63,64,65,66,67,68,69,70,71,72,73,74,75,76,77,78,82,83,84,85], four on dental surgeons [55,56,62,70], four on nurses [2,79,80,86], and two on other health-care workers [70,87]. Among those 55 on physicians, 47 reported data on deaths by suicide [1,5,7,8,15,16,34,35,36,37,38,39,40,41,42,43,44,45,46,47,48,49,50,51,52,53,54,55,56,57,58,59,60,61,62,63,64,65,66,67,68,69,70,71,72,82,83], five on suicide attempts [47,73,75,77,85], and seven on suicidal ideation [74,75,76,77,78,84,85]. In those 47 articles on deaths by suicide among physicians, 25 described SMR for suicide [7,8,41,46,52,54,55,56,57,58,59,60,61,62,63,64,65,66,67,68,69,70,71,72,82], eight reported percentages of suicide by specialty [15,16,40,43,45,47,51,83], 12 reported the number of physicians died by suicide among all deaths in physicians [16,39,41,42,44,46,48,49,50,51,52,53], and nine reported the number of physicians died by suicide among all the deaths by suicide in the general population [1,5,15,34,35,36,37,38,82]. As there are few exploitable studies about dental surgeons, nurses and other health-care workers, we won’t treat them in that meta-analysis.

More details on study characteristics (Table 1), quality of articles (Figs 2 and 3), method of sampling for markers analysis, inclusion and exclusion criteria, characteristics of participants, outcomes and aims of the studies, and study designs of included articles are described in S1 Appendix.

**Table 1:** Characteristics of included studies. CI, Confidence Interval; n, Number; SMR, Standardized Mortality Ratio; USA, United States of America.

			Time Period	Total		Suicides								
Study	Country	Continent		Physicians–n (%)		Death–n (%)		Mortality–SMR (95CI)		Attempts—n		Thoughts—n		Specialities
				Men	Women	Men	Women	Men	Women	Men	Women	Men	Women	
Aasland 2001	Norway	Europe	1960–1993			73 (89)	9 (11)							No specified
Aasland 2011	Norway	Europe	1960–2000											No specified
Arnetz 1987	Sweden	Europe	1961–1970			32 (76)	10 (24)	1,2 (0.85, 1.69)	5,7 (1.68, 10.7)					No specified
Austin 2013	Australia	Australia, New-Zealand and Pacific	1997–2011			6 (66)	3 (34)							Anaesthesiologists, psychiatrists, general practitioners, general surgeons
Bamayr 1986	Germany	Europe	1963–1978			67 (71)	27 (29)	1,58 (1.07, 2.34)	2,96 (1.44, 6.09)					No specified
Brooks 2017	USA	North America	2003–2014	1188 (72)	544 (28)							38	32	No specified
Carpenter 1997	Great Britain	Europe	1962–1979			56 (87)	8 (13)	0,96 (0.72, 1.25)	2,15 (0.93, 4.23)					No specified
Craig 1968	USA	North America	1965–1967			211	17							No specified
Davidson 2018	USA	North America	2005–2015					2,29 (1.66, 3.08)	2,29 (1.66, 3.08)					No specified
Dean 1969	South Africa	Africa	1960–1966			22 (96)	1 (4)	1,26 (0.74, 2.13)						No specified
Desole 1969	USA	North America	1965–1968											General practitioners, general surgeons, internal medicine, psychiatrists, obstetricians, anaesthesiologists, pathology, paediatrics, radiology, internships
Everson 1975	USA	North America	1966–1970											No specified
Frank 1999	USA	North America	1993–1994	0	4501 (100)						61			No specified
Frank 2000	USA	North America	1984–1995			379 (91)	37 (9)	1,7 (1.53, 1.88)	2,38 (1.69, 3.28)					No specified
Fridner 2009	Sweden and Italy	Europe	2005–2005	0	385 (100)								122	No specified
Gagne 2011	Quebec	North America	1992–2009			29 (80)	7 (20)							General practitioners, radiology, psychiatrists
Gold 2013	USA	North America	2003–2008											No specified
Gunnarsdottir 1995	Iceland	Europe	1920–1979											No specified
Hawton 2001	Great Britain	Europe	1991–1995			42 (74)	15 (26)	0,67 (0.47, 0.87)	2,02 (1.00, 3.04)					No specified
Hawton 2002	England and Wales	Europe	1994–1997											No specified
Hawton 2011	Danish	Europe	1981–2006			131 (80)	32 (20)							No specified
Hem 2000	Norway	Europe	1993–1999	722 (72)	282 (28)					7	9	61	43	No specified
Hem 2005	Norway	Europe	1960–1990			98 (88)	13 (22)							No specified
Hemenway 1993	USA	North America	1976–1988											No specified
Herner 1993	Sweden	Europe	1989–1991			17 (68)	8 (32)	1.1 (0.8, 1.52)	2,32 (1.12, 4.81)					No specified
Hikiji 2014	Japan	Asia	1996–2010			68 (79)	19 (21)							Internal medicine, dermatologists, paediatrics, psychiatrists, general surgeons, orthopaedists, ophthalmology, plastic surgeons, ENT, obstetricians, radiology, anaesthesiologists
Hubbard 1922	USA	North America	1921											No specified
Innos 2002	Estonia	Europe	1983–1998			6 (54)	5 (46)	0,58 (0.21, 1.27)	0,62 (0.20, 1.45)					No specified
Jones 1977	USA	North America	1967–1975							11	5			General practitioners, anaesthesiologists, internal medicine, obstetricians, psychiatrists, general surgeons, internships
Juel 1999	Danish	Europe	1973–1992			168 (86)	26 (14)	1.64 (1.40, 1.91)	1.68 (1.10, 2.46)					No specified
Lew 1976	USA	North America	1954–1976											No specified
Linde 1981	USA	North America	1930–1946	274 (100)	0	10	0							No specified
Lindeman 1997	Finland	Europe	1986–1993											No specified
Lindeman 2007	Finland	Europe	1987–1988			2 (28)	5 (72)							No specified
Lindfors 2009	Finland	Europe	2004–2008	175 (53)	153 (47)									No specified
Lindhardt 1963	Denmark	Europe	1935–1959					1.53 (1.06, 2.20)						No specified
Loas 2018	Belgium	Europe	2015–2018	223 (40)	334 (60)					5	9	42	91	No specified
No Author 1986	USA	North America	1980–1981											No specified
Nordentoft 1988	Netherlands	Europe	1970–1980			59 (85)	10 (15)	2.46 (1.02, 3.42)	3.33 (0.42, 26.3)					No specified
Olkinuora 1990	Finland	Europe	1986–1989	1582 (59)	1062 (41)					10	6	340	269	No specified
Palhares-Alves 2015	Brazil	South America	2000–2009			38 (76)	12 (24)							No specified
Petersen 2008	USA	North America	1984–1992			181 (89)	22 (11)	0.8 (0.53, 1.20)	2.39 (1.52, 3.77)					No specified
Pitts 1979	USA	North America	1967–1972		751		49		3.57 (1.23, 10.4)					No specified
Rafnsson 1998	Island	Europe	1955–1995			7 (100)		1.01 (0.40, 2.04)						No specified
Revicki 1985	USA	North America	1978–1982			13		1.16 (0.80, 1.70)						No specified
Rich 1979	USA	North America	1967–1972	17979		544		1.03 (0.74, 1.45)						No specified
Rich 1980	USA	North America	1967–1972			544 (92)	49 (8)							General practitioners, internal medicine, general surgeons, psychiatrists, obstetricians, paediatrics, radiology, anaesthesiologists, pathology, ophthalmology, orthopaedists
Rimpela 1987	Finland	Europe	1971–1980			17		1.28 (1.01, 1.65)						No specified
Rose 1973	USA	North America	1959–1961			48 (98)	1 (2)	2.03 (1.29, 3.19)						No specified
Roy 1985	USA	North America	1981–1974											No specified
Samkoff 1995	USA	North America	1980–1988											General practitioners, internal medicine, general surgeons, radiology, paediatrics
Schlicht 1990	Australia	Australia, New-Zealand and Pacific	1950–1986	1279 (88)	174 (12)	10	3	1.13 (0.54, 2.07)	5.01 (1.01, 14.7)					No specified
Shang 2011	Taiwan	Australia, New-Zealand and Pacific	1990–2006											No specified
Shang 2012	Taiwan	Asia	1990–2006											No specified
Simon 1968	USA	North America	1947–1967											No specified
Stefansson 1991	Sweden	Europe	1971–1985			113 (82)	25 (19)	1.82 (1.19, 2.80)	5.02 (1.67, 15.0)					No specified
Torre 2005	USA	North America	1948–1998	183 (91)	18 (11)	20 (90)	2 (10)	1.82 (1.11, 2.82)	4.95 (0.56, 17.9)					No specified
Ullmann 1991	USA	North America	1910–1981			46		1.48 (0.97, 2.27)						No specified
Wang 2017	China	Asia	2004–2017			6 (33)	8 (44)							Dermatologists, emergency, internal medicine, obstetricians, paediatrics, cardiology, neurology, urology, ophthalmology, anaesthesiologists

### Meta-analysis of the standardized mortality rate for suicides among physicians

We included 25 studies. The overall SMR was 1.44 (95CI 1.16, 1.72) with an important heterogeneity (I^2^ = 93.9%). Among the 25 included studies, 17 studies reported both male and female physicians [7,8,41,46,52,54,55,56,57,58,59,61,62,68,70,71,82], six reported only male physicians [60,64,65,66,67,72], and one only reported female physicians [63]. We found a significantly higher risk of suicide among male physicians than in the general population (SMR = 1.24; 95CI 1.05, 1.43; *P* < 0.001; I^2^ = 79.1%) and for suicide among female physicians than in the general population (SMR = 1.94; 95CI 1.49, 2.58; *P* < 0.041; I^2^ = 42.5%) (Fig 4). Meta-regressions demonstrated that women physicians had a higher risk than their counterpart men to commit suicide (0.67; 95CI 0.19, 1.14; *P* = 0.007) (Fig 5). We further demonstrated that the risk of suicide was not homogeneous over all the countries. SMR was 1.27 (95CI 1.05, 1.49; *P* < 0.001; I^2^ = 71.3%) in Europe, 1.63 (95CI 1.29, 1.96; *P* < 0.001; I^2^ = 74.1%) in North America, 0.79 (95CI 0.03, 1.62; *P* = 0.002; I^2^ = 79.5%) in Australia, New-Zeeland and Pacific and 1.26 (95CI 0.56, 1.96) in Africa (Fig 6). Meta-regressions demonstrated a higher risk of suicide in North America than in Australia, New-Zeeland and Pacific (0.92; 95CI 0.22, 1.63; *P* = 0.013) and especially higher in USA vs the rest of the world (1.34; 95CI 1.28, 1.55; *P* < 0.001) (Fig 5).

Finally, we demonstrated an overall time effect (-0.15; 95CI -0.29, -0.01; *P* = 0.032) which signify that the risk decreased over time. This relationship is significant in Europe (-0.18; 95CI -0.37, -0.01; *P* = 0.044) but not in USA (-0.11; 95CI -0.37, 0.15; *P* = 0.370) or in Australia, New-Zeeland and Pacific (-0.48; 95CI -8.09, 7.12; *P* = 0.570). For Africa, there were insufficient observations (Fig 5).

### Meta-analysis of percentage of suicide in physicians by group of specialties

We included eight studies [15,16,40,43,45,47,51,83]. The percentage of suicide in general practitioners was 32% (95CI 21, 43; *P* < 0.001; I^2^ = 93.1%), in internal medicine was 16% (95CI 9, 23; *P* < 0.001; I^2^ = 88.6%), in psychiatrists was 11% (95CI 9, 14; *P* = 0.30; I^2^ = 17.5%), in other medical specialties was 3% (95CI 3, 4; *P* = 0.02; I^2^ = 40.7%), in surgeons was 4% (95CI 2, 5; *P* < 0.001; I^2^ = 62.8%) and in internships was 2% (95CI 1, 4) (Fig 7).

Meta-regressions demonstrated a higher risk of suicide in general practitioners than internal medicine (0.12; 95CI 0.05, 0.19; *P* = 0.001), than psychiatrists (0.17; 95CI 0.09, 0.24; *P* < 0.001), than other medical specialties (0.24; 95CI 0.18, 0.30; *P* < 0.001), than surgeons (0.25; 95CI 0.19, 0.30; *P* < 0.001) and then internships (0.24; 95CI 0.15, 0.34; *P* < 0.001). Moreover, a higher risk of suicide in internal medicine than in other medical specialties (0.12; 95CI 0.08, 0.17; *P* < 0.001), than surgeons (0.13; 95CI 0.08, 0.18; *P* < 0.001), and than internships (0.13; 95CI 0.03, 0.22; *P* = 0.008). Finally, we demonstrated a higher risk of suicide in psychiatrists than other medical specialties (0.07; 95CI 0.02, 0.13; *P* = 0.009) and than surgeons (0.08; 95CI 0.02, 0.13; *P* = 0.005) (S1 Fig).

### Meta-analysis of percentages of suicide in physicians by category of surgical specialties

We included six studies [15,16,43,47,51,83]. The percentage of suicide in general surgeons was 6% i.e. (95CI 4, 9; I^2^ = 64.5%, *P =* 0.04*)*, in obstetricians was 4% (95CI 2, 5; I^2^ = 0, *P =* 0.81*)*, in orthopaedists was 2% (95CI 1, 4), in ears, nose and throat was 3% (95CI 0, 3) and in plastic surgeons was 1% (95CI 0, 6) (Fig 8).

Meta-regressions demonstrated a higher risk of suicide in general surgeons than obstetricians (0.03; 95CI 0.01, 0.05; *P* = 0.035), than orthopedists (0.04; 95CI 0.01, 0.07; *P* = 0.006), than ophthalmologists (0.04; 95CI 0.02, 0.07; *P* = 0.006) and than plastic surgeons (0.05; 95CI 0.01, 0.09; *P* = 0.010) (S2 Fig).

### Meta-analysis of percentages of suicide in physicians by category of medical specialties

Eight studies were included [15,16,40,43,45,47,51,83]. The percentage of suicide in internal medicine was 16% (95CI 9, 23; I^2^ = 88.6%, *P* < 0.001), in psychiatrists was 11% (95CI 9, 14; I^2^ = 17.5%, *P* = 0.30), in anaesthesiologists was 4% (95CI 2, 6; I^2^ = 43.6%, *P* = 0.11), in radiologists was 3% (95CI 2, 5; I^2^ = 66.0%, *P* = 0.02), in paediatricians was 4% (95CI 3, 6; I^2^ = 46.4%, *P* = 0.11), in pathologists was 2% (95CI 1, 3), in dermatologists was 5% (95CI 1, 9), in cardiologists was 6% (95CI 1, 26), in neurologists was 6% (95CI 1, 26) and in emergency physicians was 6% (95CI 1, 26) (Fig 9). Meta-regressions demonstrated a higher risk of suicide in internal medicine than anesthesiologists (0.12; 95CI 0.06, 0.18; *P* = 0.001) than radiologists (0.13; 95CI 0.07, 0.19; *P* < 0.001), than pediatricians (0.12; 95CI 0.06, 0.18; *P* = 0.001) than pathologists (0.14; 95CI 0.07, 0.21; *P* < 0.001) and than dermatologists (0.12; 95CI 0.03, 0.21; *P* = 0.13). Moreover, the risk of suicide was higher in psychiatrists than anesthesiologists (0.07; 95CI 0.01, 0.13; *P* = 0.038), than radiologists (0.08; 95CI 0.02, 0.14; *P* = 0.014), than pediatricians (0.07; 95CI 0.01, 0.13; *P* = 0.038) and than pathologists (0.09; 95CI 0.02, 0.17; *P* = 0.014) (S3 Fig).

### Meta-analysis of prevalence of physicians dead by suicide among all deaths in physicians

We included 12 studies [16,39,41,42,44,46,48,49,50,51,52,53], and we demonstrated a prevalence of 4% (95CI 3, 5) with an important heterogeneity (I^2^ = 88.7%) (Fig 10).

Meta-regression on geographic zones did not retrieves any significant result. Moreover, insufficient data did not permit other meta-regression.

### Meta-analysis of the prevalence of deaths by suicide in physicians among all deaths by suicide in the general population

We included nine studies [1,5,15,34,35,36,37,38,82], and we demonstrated a prevalence of 1% (95CI 1, 1) with an important heterogeneity (I^2^ = 98.0%) (S4 Fig). Insufficient data did not permit meta-regression.

### Meta-analysis of the number of physicians having done suicide attempt among all the physicians

We included five studies [47,57,75,77,85]. The overall effect size was 0.01 (95CI 0.01, 0.02; p < 0.01) with an important heterogeneity (I^2^ = 82.6%) (S5 Fig). Insufficient data did not permit meta-regression.

### Meta-analysis of the number of physicians with suicidal ideation among all the physicians

We included seven studies [74,75,76,77,78,84,85]. The overall effect size was 0.17 (95CI 0.12, 0.21; p < 0.001) with an important heterogeneity (I^2^ = 98.8%) (Fig 11). Insufficient data did not permit meta-regression.

### Other health care workers

As there are few exploitable studies about dental surgeons, nurses and other health-care workers, we didn’t treat them in that meta-analysis.

## Discussion

Physicians were an at-risk profession (1.44, 95CI 1.16, 1.72), particularly women-physician (0.67, 95CI 0.19, 1.14; p = 0.007). Some countries had a high risk of suicide (USA vs Rest of the world: 1.34, 95CI 1.28, 1.55; p < 0.001) and rate of suicide in physicians decreased over time, especially in Europe (-0.18, 95CI -0.37, -0.01; p = 0.044). Some specialties were higher risk such as anesthesiologists, psychiatrists, general practitioners and general surgeons. The prevalence of physicians having done suicide attempt among all the physicians were significant (0.01, 95CI 0.01, 0.02; p < 0.001) as the prevalence of physicians with suicidal ideation among all the physicians (0.17, 95CI 0.12, 0.21; p < 0.001). Finally, there were not enough exploitable data about dental surgeons, nurses and other health-care workers which are however some at-risk professions.

### An at-risk profession

The high risk of suicide in physicians might be explained by several putative factors such as psychosocial working environment [18], or specific personality traits of physicians. Psychosocial work environment has been shown in the literature as an important risk factor, doctors being confronted to conflicts with colleagues, lack of cohesive teamwork and social support, leading them individually [88]. Physicians must also routinely face with breaking bad news [89], and are in frequent contact with illness, anxiety, suffering and death. Perfectionism, compulsive attention to detail, exaggerated sense of duty, excessive sense of responsibility, desire to please everyone are appreciates qualities in workplace [90,91] but increased stress and depression [92] and imprison physicians in vicious circle without seek help. They also prevent themselves to ask for help because of the culture of medical education [90,91]. In particular, we demonstrated that women physicians were particularly exposed to suicide, which might be explained by the additional strain imposed on them because of their social roles [11]. In most countries, women still have more at-home responsibilities (education of children, nursing, household care, etc) than men. Combining a full-time job as a physician and those at-home responsibilities might be particularly difficult to manage [11]. Although income gender-inequalities have not been reported in physicians[93,94], some authors suggested that the medical field was mainly dominated by the male gender and reported a poor status integration of women physicians within the profession [7]. It has been shown that female physicians/internships react by imposing themselves an additional pressure to demonstrate their male counterparts that they are as strong, self-sufficient and worthy as them [95].

### Depending on countries

We showed that the risk of suicide was not homogeneous between countries, in line with inequality of job satisfaction among physicians in many countries [96,97]. Indeed, some countries such as Switzerland and Canada reported a high level of job satisfaction for physicians (>75%) [98,99]. In the United States, most obstetrician gynecologists only rated their job satisfaction as moderate [100]. Physician job satisfaction is essential for ensuring the quality and sustainability of health care provision [101,102]. Moreover, career dissatisfaction was associated with burnout and prolonged fatigue among physicians [103]. In most countries, physicians’ work conditions underwent frequent mutations, with multiple healthcare reforms initiatives promoting by local governments. Reforms are a necessary compromise between best outcomes on deliveries of care, health economics, and quality of work environment [104,105].

### With a time effect

There are few data on the evolution of the rate of suicide over time and we were the first to demonstrate that, in some countries such as in Europe the suicide rate among physicians decreased significantly with time but not in the USA. During the past decade, a confluence of forces has changed the practice of medicine in unprecedented ways. Indeed, physicians have seen their autonomy reduced by increased administrative tasks and time pressure [106,107,108]. In USA, a survey showed that physicians’ satisfaction declined over the last 10 years, with less time spent per patient and for private life [13]. US physicians might also be particularly stress [109] because of medical errors that are the third leading cause of death in US [110,111] in a context of economic pressure and relationships with pharmaceutic companies [112,113], religious beliefs [114], access care difficulties for some patients [115], and legal procedure intended against physicians [116] leading them to practice a more defensive medicine [117] misleading patients in overdiagnosis [118]. The World Health Organization global strategy on human resources for health (workforce 2030) promoted the personal and professional rights of health-care workers, including safe and decent working environments [119]. Particularly in Europe, working hours of physicians decreased significantly over the last decades following official instructions such as the European Working Time Directive (EWTD) [14], which may have contributed to a decreased risk of suicides.

### Some specialties are more at-risk

We showed some the most at-risk specialties were anaesthesiologists, psychiatrists, general practitioners and general surgeons. The high risk of suicides in anaesthesiologists [16,41,48,76] could be explained by an easy access to potentially lethal drugs, a high prevalence of burnout [120], a high workload with fear of harming patients and organizational burden with poor autonomy, and conflicts with colleagues [121]. For psychiatrists, the high risk of suicides has been linked by stressful and traumatic experiences such as, paradoxically, dealing with suicides of patient [16]. Next to those medical specialties, the general practitioners were an historical at-risk occupation, with moral loneliness, job interfering with family life, constant interruptions both at home and at work, increasing administrative constraints, and high levels of patients' expectations, leading to a low job satisfaction and poor mental health [122,123]. Finally, specialties with life-and-death emergencies, like surgery, are particularly stressful [124,125,126,127]. For example, it has been shown that intra-operative death increased morbidity in patients operated by the same surgeon in the subsequent 48 hours, with a more pronounced whether the death occurring during emergency surgery [128].

### Suicide attempts and suicidal ideation

Suicide could be regarded as a lengthy process. Little is known about causes and transitions between suicidal ideation / attempted suicide and suicide, as well as about the factors that precipitate or protect against these transitions [129]. Because physicians might be more aware of these characteristics than the general population [75], having suicidal thoughts should be taken particularly seriously in this profession. Suicidal ideation are considered a sensitive and specific indicator of suicide risk [130,131]. Preventive strategies may include improved management of psychiatric disorders, the recognition and treatment of depression and substances abuse [65], but also measures to reduce occupational stress, and restriction of access to means of suicide when doctors are depressed [4,132]. Medical school curriculum should also include programs to increase students’ self-confidence, to express their emotional needs, and to teach that anyone may be suicidal–regardless of his status [133]. The preventive approach may consist of screening, assessment, referral and education, and to destigmatize help-seeking at-risk medical students/physicians [134].

### Suicides in other health-care workers

We highlighted the lack of studies providing data on deaths by suicide and on suicidal risks in nurses and in other health-care workers. However, nurses remained at high-risk of suicide with various stressful factors comparable to those previously described for physicians, such as patients cares, team’s conflicts, heavy workload, lack of autonomy, and work-family conflicts [135,136]. As for physicians, some occupational settings were described as particularly stressful, such as working in emergency departments [137], with a high prevalence of shift work [138], exposure to aggressive and violent behavior from patients [139] and from situation relating to trauma, alcohol and intoxications [140]. Our study demonstrated the lack of data on other health-care workers such as pharmacists, dental surgeons, midwives, caregivers and hospital maids. We believe that such data are needed.

### Limitations

Our study has however some limitations. Meta-analyses inherit the limitations of the individual studies of which they are composed: varying quality of studies and multiple variations in study protocols and evaluation. We highlighted that general practitioners were prone to suicide. However, comparisons between specialties may suffer from a major bias such as different number of physicians within each specialty (not the same denominator in statistical analyses—there are more suicides among general practitioners because there are more general practitioners than other individual specialties). All included studies on death by suicide in physicians were retrospective and based on health registers, and thus few studies reported details on occupation such as seniority or characteristics of practice, precluding further analyses necessary for effective preventive strategies. The studies on suicide attempts and suicidal ideation that were based on self-report questionnaire [73,74,75,77] may lack of standardized interviews or specifics criteria for diagnoses psychiatric disorders [125,[141]. Most cross-sectional studies included in our meta-analyses described a bias of self-report such as skipping questions and incomplete information, nondisclosure, and uncertainty regarding timing of questionnaire. Percentage of respondents within those studies may seem low, from 45% [74] to 76% [77], however the response rate was higher than usual [142,143,144,145,146]. The language used in countries with two official languages may also have influenced responses [74]. Only one study questioned physicians on their antidepressant treatment [121], and only one study questioned about a psychiatric disorder [74]. More data is needed regarding physician’s health. Finally, none of the studies included specified whether some physicians were retired or not.

## Conclusion

Preventive strategies on the risk of suicides in physicians are strongly needed. Physicians are an at-risk profession of suicide, with a global SMR of 1.44 (95CI 1.16, 1.72), and an important heterogeneity between studies. Women were particularly at risk compared to male physicians. In addition, some countries were with a higher risk of suicide such as USA. Interestingly, the rate of suicide in physicians decreased over time, especially in Europe, suggesting improvements of working conditions of physicians. Some specialties might be at higher risk such as anesthesiologists, psychiatrists, general practitioners and general surgeons. The high prevalence of physicians who committed suicide attempts as well as those with suicidal ideation should benefits for preventive strategies at the workplace. Public health policies must aim at improving social work environment and contribute to screening, assessment, referral, and destigmatization of suicides in physicians. Finally, the lack of data on other health-care workers suggest implementing studies investigating those occupations who might also be at risk of suicide.

## Supporting information

S1 Appendix Details on study characteristics, quality of articles (Figs 2 and 3), method of sampling for markers analysis, inclusion and exclusion criteria, characteristics of participants, outcomes and aims of the studies, and study designs of included articles. (DOCX)

S2 Appendix PRISMA checklist. (DOCX)

S1 Fig Meta-regression of percentages of suicide in physicians by group of specialties. (TIF)

S2 Fig Meta-regression of percentages of suicide in physicians by category of surgical specialties. (TIF)

S3 Fig Meta-regression of percentages of suicide in physicians by category of medical specialties. (TIF)

S4 Fig Meta-analysis of prevalence of physicians died by suicide among all the deaths by suicide in the general population. (TIF)

S5 Fig Meta-analysis of prevalence of physicians having done suicide attempt among all the physicians. (TIF)
