# Supplementary material for: Suicide among physicians and health-care workers: A systematic review and meta-analysis
Source: PLoS One. 2019 Dec 12;14(12):e0226361. doi: 10.1371/journal.pone.0226361 (PMC6907772; doi:10.1371/journal.pone.0226361)
Supplement: S1 Appendix — (DOCX) [file pone.0226361.s001.docx]

**S1 Appendix**. **Details on study characteristics, quality of articles (Fig1 and 2), method of sampling for markers analysis, inclusion and exclusion criteria, characteristics of participants, outcomes and aims of the studies, and study designs of included articles**.

The article already described studies on physicians. For studies reporting dental surgeons, two studies reported the number of dental surgeons died by suicide among all deaths in dental surgeons [[55](#_ENREF_55),[56](#_ENREF_56)], and two studies reported SMR for suicides in dental surgeons [[62](#_ENREF_62),[70](#_ENREF_70)]. For studies about nurses, one study reported the number of nurses died by suicide among all deaths in nurses [[2](#_ENREF_2)], and two studies reported the number of nurses died by suicide among all nurses in the general population [[79](#_ENREF_79),[80](#_ENREF_80)]. For studies about other health-care workers, one study reported the SMR for suicides in auxiliary nurses [[70](#_ENREF_70)], and one study reported the SMR for suicide in psychologists [[87](#_ENREF_87)].

**Quality of articles**

All included studies, except one [[37](#_ENREF_37)], were observational and their quality assessment was performed using the STROBE and NOS criteria, respectfully:

**- SMR for suicides among physicians:** 25 studies, a mean score of 26.5/30 (88%) (varying from 17/30 (57%) [[66](#_ENREF_66)] to 28/30 (93%)) [[7](#_ENREF_7),[57](#_ENREF_57)], and a mean score of 8.7/9 (97%) (varying from 8/9 (89%) [[41](#_ENREF_41),[46](#_ENREF_46),[54](#_ENREF_54),[55](#_ENREF_55),[56](#_ENREF_56),[60](#_ENREF_60),[68](#_ENREF_68),[82](#_ENREF_82)] to 9/9 (100%) [[7](#_ENREF_7),[8](#_ENREF_8),[52](#_ENREF_52),[57](#_ENREF_57),[59](#_ENREF_59),[62](#_ENREF_62),[63](#_ENREF_63),[65](#_ENREF_65),[66](#_ENREF_66),[67](#_ENREF_67),[69](#_ENREF_69),[70](#_ENREF_70),[71](#_ENREF_71),[72](#_ENREF_72)])

**- Percentages of suicide by specialties:** 8 studies, a mean score of 19/30 (63%) (varying from 15/30 (50%) [[43](#_ENREF_43),[47](#_ENREF_47)] to 24/30 (80%)) [[45](#_ENREF_45)], and a mean score of 7.6/9 (84%) (varying from to 6/9 (67%) [[40](#_ENREF_40),[43](#_ENREF_43),[47](#_ENREF_47),[83](#_ENREF_83)] to 9/9 (100%) [[15](#_ENREF_15),[45](#_ENREF_45),[51](#_ENREF_51)]).

**- Physicians dead by suicide among all deaths in physicians:** 12 studies, a mean score of 21/30 (70%) (varying from 15/30 (50%) [[88](#_ENREF_88)] to 25/30 (83%)) [[50](#_ENREF_50),[52](#_ENREF_52)], and a mean score of 8.2/9 (91%) (varying from 7/9 (78%) [[44](#_ENREF_44),[53](#_ENREF_53),[88](#_ENREF_88)] to 9/9 (100%) [[42](#_ENREF_42),[49](#_ENREF_49),[50](#_ENREF_50),[51](#_ENREF_51),[52](#_ENREF_52)].

**- Physicians dead by suicide among all deaths by suicide in the general population:** 9 studies, a mean score of 22/30 (74%) (varying from 19/30 (63%) [[15](#_ENREF_15)] to 25/30 (83%)) [[35](#_ENREF_35),[36](#_ENREF_36)], and a mean score of 8.7/9 (97%) (varying from 7/9 (0%) [[89](#_ENREF_89)] to 9/9 (100%) [[1](#_ENREF_1),[15](#_ENREF_15),[34](#_ENREF_34),[35](#_ENREF_35),[36](#_ENREF_36),[82](#_ENREF_82),[90](#_ENREF_90)]).

**- Physicians having done suicide attempt among all the physicians:** 5 studies, a mean score of 18.25/30 (61%) (varying from 15/30 (50%) [[47](#_ENREF_47)] to 20/30 (66%)) [[73](#_ENREF_73)], and a mean score of 5.8/9 (64%) (varying from 4/9 (44%) [[73](#_ENREF_73),[75](#_ENREF_75),[85](#_ENREF_85)] to 9/9 (100%) [[77](#_ENREF_77)].

**- Physicians with suicidal ideation among all the physicians:** 7 studies, a mean score of 23.4/30 (78%) (varying from 17/30 (63%) [[78](#_ENREF_78)] to 22/30 (83%)) [[74](#_ENREF_74)], and a mean score of 5.6/9 (62%) (varying from 4/9 (44%) [[74](#_ENREF_74),[75](#_ENREF_75),[85](#_ENREF_85)] to 9/9 (100%) [[77](#_ENREF_77),[84](#_ENREF_84)].

Moreover, for 1 study and for 5 studies, STROBE [[37](#_ENREF_37)] and NOS [[37](#_ENREF_37),[58](#_ENREF_58),[61](#_ENREF_61),[81](#_ENREF_81),[91](#_ENREF_91)] criteria couldn't be applied, respectfully.

**Method of sampling for markers analysis**

For studies reporting **Standardized Mortality Rate for suicides**, databases used were national registers of mortality in the general population for 20 studies [[7](#_ENREF_7),[8](#_ENREF_8),[41](#_ENREF_41),[46](#_ENREF_46),[52](#_ENREF_52),[54](#_ENREF_54),[57](#_ENREF_57),[58](#_ENREF_58),[59](#_ENREF_59),[60](#_ENREF_60),[61](#_ENREF_61),[62](#_ENREF_62),[63](#_ENREF_63),[65](#_ENREF_65),[68](#_ENREF_68),[69](#_ENREF_69),[70](#_ENREF_70),[71](#_ENREF_71),[72](#_ENREF_72),[82](#_ENREF_82)], registers only within medical occupations for four studies [[7](#_ENREF_7),[52](#_ENREF_52),[55](#_ENREF_55),[56](#_ENREF_56)], deaths recorded by associations for five studies [[8](#_ENREF_8),[52](#_ENREF_52),[63](#_ENREF_63),[66](#_ENREF_66),[72](#_ENREF_72)], specific registers like statistic registers for six studies [[8](#_ENREF_8),[46](#_ENREF_46),[52](#_ENREF_52),[56](#_ENREF_56),[64](#_ENREF_64),[67](#_ENREF_67)], and register of medicolegal autopsies for one study [[8](#_ENREF_8)]. Data were collected mainly from one database for 14 studies [[55](#_ENREF_55),[57](#_ENREF_57),[58](#_ENREF_58),[60](#_ENREF_60),[61](#_ENREF_61),[62](#_ENREF_62),[64](#_ENREF_64),[65](#_ENREF_65),[66](#_ENREF_66),[67](#_ENREF_67),[68](#_ENREF_68),[69](#_ENREF_69),[70](#_ENREF_70),[71](#_ENREF_71)], from two databases for five studies [[46](#_ENREF_46),[54](#_ENREF_54),[56](#_ENREF_56),[59](#_ENREF_59),[63](#_ENREF_63)], from three databases for three studies [[7](#_ENREF_7),[41](#_ENREF_41),[72](#_ENREF_72)], from four databases for one study [[8](#_ENREF_8)], and from seven databases for one study [[52](#_ENREF_52)].

For studies reporting **percentages of suicide by specialties**, databases used were national registers of mortality in the general population for one study [[43](#_ENREF_43)], registers only within medical occupations for three studies [[15](#_ENREF_15),[40](#_ENREF_40),[45](#_ENREF_45),[83](#_ENREF_83)], deaths recorded by associations for two studies [[16](#_ENREF_16),[51](#_ENREF_51)], specific registers like the hospital case records for one study [[47](#_ENREF_47)]. Data were collected mainly from one database for the 7 studies [[15](#_ENREF_15),[16](#_ENREF_16),[40](#_ENREF_40),[43](#_ENREF_43),[45](#_ENREF_45),[47](#_ENREF_47),[51](#_ENREF_51)].

For studies reporting **the number of physicians dead by suicide among all deaths in physicians,** databases used were national registers of mortality in the general population for four studies [[41](#_ENREF_41),[46](#_ENREF_46),[50](#_ENREF_50),[52](#_ENREF_52)], registers only within medical occupations for two studies [[48](#_ENREF_48),[49](#_ENREF_49)], deaths recorded by associations for six studies [[16](#_ENREF_16),[42](#_ENREF_42),[44](#_ENREF_44),[51](#_ENREF_51),[53](#_ENREF_53),[88](#_ENREF_88)], and specific registers like statistic registers for two studies [[46](#_ENREF_46),[52](#_ENREF_52)]. Data were collected mainly from one database for seven studies [[16](#_ENREF_16),[42](#_ENREF_42),[44](#_ENREF_44),[48](#_ENREF_48),[51](#_ENREF_51),[53](#_ENREF_53),[88](#_ENREF_88)], two databases for three studies [[46](#_ENREF_46),[49](#_ENREF_49),[50](#_ENREF_50),[54](#_ENREF_54),[56](#_ENREF_56),[59](#_ENREF_59),[63](#_ENREF_63)], three databases for one study [[41](#_ENREF_41)], and seven databases for one study [[52](#_ENREF_52)].

For studies reporting **the number of deaths by suicide in physicians among all deaths by suicide in the general population,** databases used were national registers of mortality in the general population for four studies [[1](#_ENREF_1),[5](#_ENREF_5),[37](#_ENREF_37),[38](#_ENREF_38),[82](#_ENREF_82)], registers only within medical occupations for two studies [[15](#_ENREF_15),[34](#_ENREF_34)], specific registers like statistic registers for three studies [[5](#_ENREF_5),[34](#_ENREF_34),[35](#_ENREF_35)], and like national violent death register for one study [[36](#_ENREF_36)]. Data were collected mainly from one database for six studies [[1](#_ENREF_1),[15](#_ENREF_15),[35](#_ENREF_35),[36](#_ENREF_36),[37](#_ENREF_37),[38](#_ENREF_38)], and two databases for two studies [[5](#_ENREF_5),[34](#_ENREF_34)].

For the studies reporting **the number of physicians having done suicide attempt among all the physicians,** databases used were deaths recorded by associations for three studies [[73](#_ENREF_73),[75](#_ENREF_75),[77](#_ENREF_77)], specific registers like the hospital case records for one study [[47](#_ENREF_47)]. Data were collected mainly from one database for four studies [[47](#_ENREF_47),[57](#_ENREF_57),[75](#_ENREF_75),[77](#_ENREF_77)].

For the studies reporting **the number of physicians with suicidal ideation among all the physicians,** databases used were deaths recorded by associations for three studies [[75](#_ENREF_75),[76](#_ENREF_76),[77](#_ENREF_77)], specific registers like a specific university case records for two studies [[74](#_ENREF_74),[78](#_ENREF_78)]. Data were collected mainly from one database for four studies [[75](#_ENREF_75),[76](#_ENREF_76),[77](#_ENREF_77),[78](#_ENREF_78)], and from two databases for one study [[74](#_ENREF_74)].

In addition, for the studies about suicide attempts and suicidal ideation, seven of the nine included studies also used a **questionnaire** [[57](#_ENREF_57),[74](#_ENREF_74),[75](#_ENREF_75),[76](#_ENREF_76),[77](#_ENREF_77),[84](#_ENREF_84),[85](#_ENREF_85)]. A paper questionnaire was sent by postal mail in four studies [[73](#_ENREF_73),[75](#_ENREF_75),[76](#_ENREF_76),[77](#_ENREF_77)], and in the Sweden group of the Fridner et al. study [[74](#_ENREF_74)]. The Italian group of the Fridner et al. study [[74](#_ENREF_74)], received a postal mail containing a personal password and log-on information to access a Web-based questionnaire [[74](#_ENREF_74)]. Two of the studies didn’t use a questionnaire [[47](#_ENREF_47),[78](#_ENREF_78)].

**Inclusion criteria**

For studies on **Standardized Mortality Rate for suicides among physicians**, 15 studies included physicians and other professions [[8](#_ENREF_8),[54](#_ENREF_54),[55](#_ENREF_55),[56](#_ENREF_56),[57](#_ENREF_57),[58](#_ENREF_58),[59](#_ENREF_59),[60](#_ENREF_60),[61](#_ENREF_61),[62](#_ENREF_62),[64](#_ENREF_64),[65](#_ENREF_65),[67](#_ENREF_67),[68](#_ENREF_68),[70](#_ENREF_70)], and ten studies only included physicians [[7](#_ENREF_7),[41](#_ENREF_41),[46](#_ENREF_46),[52](#_ENREF_52),[63](#_ENREF_63),[66](#_ENREF_66),[69](#_ENREF_69),[71](#_ENREF_71),[72](#_ENREF_72),[82](#_ENREF_82)].

For studies reporting **percentages of suicide by specialties**, six studies included only physicians [[16](#_ENREF_16),[40](#_ENREF_40),[43](#_ENREF_43),[47](#_ENREF_47),[51](#_ENREF_51),[83](#_ENREF_83)], and two studies included physicians and other professions [[15](#_ENREF_15),[45](#_ENREF_45)]. Moreover, three studies included only men [[60](#_ENREF_60),[64](#_ENREF_64),[72](#_ENREF_72)], and one study included only white men and women [[62](#_ENREF_62)].

For studies on **the number of physicians dead by suicide among all deaths in physicians**, nine studies included physicians [[39](#_ENREF_39),[41](#_ENREF_41),[42](#_ENREF_42),[44](#_ENREF_44),[46](#_ENREF_46),[50](#_ENREF_50),[51](#_ENREF_51),[52](#_ENREF_52),[53](#_ENREF_53)], two studies included only anesthesiologists [[48](#_ENREF_48),[49](#_ENREF_49)], and one included only psychiatrists [[16](#_ENREF_16)]. Moreover one study included only white male [[49](#_ENREF_49)].

For studies on **the number of deaths by suicide in physicians among all deaths by suicide in the general population**, eight studies included physicians and other professions [[1](#_ENREF_1),[5](#_ENREF_5),[15](#_ENREF_15),[34](#_ENREF_34),[35](#_ENREF_35),[37](#_ENREF_37),[38](#_ENREF_38)], and two studies included only physicians [[36](#_ENREF_36),[82](#_ENREF_82)].

For the studies reporting **the number of physicians having done suicide attempt among all the physicians**, five studies included only physicians [[74](#_ENREF_74),[75](#_ENREF_75),[77](#_ENREF_77),[78](#_ENREF_78),[85](#_ENREF_85)], and one study included only anaesthesiologists [[76](#_ENREF_76)]. Moreover, one study included only women [[74](#_ENREF_74)].

For the studies reporting **the number of physicians with suicidal ideation among all the physicians**, all the studies included physicians [[74](#_ENREF_74),[75](#_ENREF_75),[76](#_ENREF_76),[77](#_ENREF_77),[78](#_ENREF_78),[84](#_ENREF_84),[85](#_ENREF_85)], and one study included only women [[73](#_ENREF_73)].

**Exclusion criteria**

Most studies (47/51 i.e. 92.1%) did not mentioned any exclusion criterion. The other studies had the following exclusion criteria: female gender [[49](#_ENREF_49)], medical students and physicians assistants [[36](#_ENREF_36)], human service educations (such as auxiliary nurses, psychologists and social workers) [[5](#_ENREF_5)], individuals of race/ethnicity other than white or black [[57](#_ENREF_57)].

**Characteristics of participants**

**Sex***:* 45 studies reported male and female physicians [[1](#_ENREF_1),[5](#_ENREF_5),[7](#_ENREF_7),[8](#_ENREF_8),[15](#_ENREF_15),[16](#_ENREF_16),[34](#_ENREF_34),[35](#_ENREF_35),[36](#_ENREF_36),[37](#_ENREF_37),[39](#_ENREF_39),[40](#_ENREF_40),[41](#_ENREF_41),[42](#_ENREF_42),[44](#_ENREF_44),[45](#_ENREF_45),[46](#_ENREF_46),[47](#_ENREF_47),[48](#_ENREF_48),[50](#_ENREF_50),[51](#_ENREF_51),[52](#_ENREF_52),[53](#_ENREF_53),[54](#_ENREF_54),[55](#_ENREF_55),[56](#_ENREF_56),[57](#_ENREF_57),[58](#_ENREF_58),[59](#_ENREF_59),[61](#_ENREF_61),[62](#_ENREF_62),[67](#_ENREF_67),[68](#_ENREF_68),[69](#_ENREF_69),[70](#_ENREF_70),[71](#_ENREF_71),[75](#_ENREF_75),[76](#_ENREF_76),[77](#_ENREF_77),[78](#_ENREF_78),[82](#_ENREF_82),[83](#_ENREF_83),[84](#_ENREF_84),[85](#_ENREF_85)], six men physicians [[49](#_ENREF_49),[60](#_ENREF_60),[64](#_ENREF_64),[65](#_ENREF_65),[66](#_ENREF_66),[72](#_ENREF_72)], three women physicians [[63](#_ENREF_63),[73](#_ENREF_73),[74](#_ENREF_74)], and for one study, sex was unspecified [[43](#_ENREF_43)].

**Age*:*** Mean age was reported in 13 studies [[15](#_ENREF_15),[16](#_ENREF_16),[36](#_ENREF_36),[45](#_ENREF_45),[50](#_ENREF_50),[53](#_ENREF_53),[57](#_ENREF_57),[63](#_ENREF_63),[66](#_ENREF_66),[71](#_ENREF_71),[76](#_ENREF_76),[84](#_ENREF_84),[85](#_ENREF_85)]. The mean age was reported for physicians who committed suicide in six studies [[15](#_ENREF_15),[16](#_ENREF_16),[45](#_ENREF_45),[50](#_ENREF_50),[63](#_ENREF_63),[66](#_ENREF_66)], for all physicians dead in four studies [[36](#_ENREF_36),[53](#_ENREF_53),[57](#_ENREF_57),[71](#_ENREF_71)], and for physicians who responded to the questionnaire for one study [[76](#_ENREF_76)]. 22 studies reported 1-5 age groups [[1](#_ENREF_1),[5](#_ENREF_5),[8](#_ENREF_8),[40](#_ENREF_40),[41](#_ENREF_41),[44](#_ENREF_44),[48](#_ENREF_48),[51](#_ENREF_51),[58](#_ENREF_58),[59](#_ENREF_59),[61](#_ENREF_61),[67](#_ENREF_67),[70](#_ENREF_70),[72](#_ENREF_72),[73](#_ENREF_73),[74](#_ENREF_74),[75](#_ENREF_75),[77](#_ENREF_77)], nine studies reported 6-10 age groups [[7](#_ENREF_7),[42](#_ENREF_42),[49](#_ENREF_49),[53](#_ENREF_53),[54](#_ENREF_54),[55](#_ENREF_55),[56](#_ENREF_56),[62](#_ENREF_62),[88](#_ENREF_88)] and four studies reported more than 10 age groups [[34](#_ENREF_34),[35](#_ENREF_35),[43](#_ENREF_43),[60](#_ENREF_60)]. Ages varied from 18 years old [[1](#_ENREF_1)] to 100 years old [[43](#_ENREF_43)]. Four studies did not mentioned age [[37](#_ENREF_37),[64](#_ENREF_64),[68](#_ENREF_68),[78](#_ENREF_78)]. Moreover, no study reported if the physicians were retired.

**Geographic Zone*:*** 23 studies reported data on European physicians [[1](#_ENREF_1),[5](#_ENREF_5),[7](#_ENREF_7),[8](#_ENREF_8),[34](#_ENREF_34),[35](#_ENREF_35),[41](#_ENREF_41),[46](#_ENREF_46),[54](#_ENREF_54),[55](#_ENREF_55),[58](#_ENREF_58),[59](#_ENREF_59),[60](#_ENREF_60),[61](#_ENREF_61),[64](#_ENREF_64),[67](#_ENREF_67),[70](#_ENREF_70),[74](#_ENREF_74),[75](#_ENREF_75),[76](#_ENREF_76),[77](#_ENREF_77),[85](#_ENREF_85)], 24 on North American physicians [[16](#_ENREF_16),[36](#_ENREF_36),[37](#_ENREF_37),[39](#_ENREF_39),[42](#_ENREF_42),[43](#_ENREF_43),[44](#_ENREF_44),[45](#_ENREF_45),[47](#_ENREF_47),[48](#_ENREF_48),[49](#_ENREF_49),[51](#_ENREF_51),[57](#_ENREF_57),[62](#_ENREF_62),[63](#_ENREF_63),[65](#_ENREF_65),[66](#_ENREF_66),[68](#_ENREF_68),[71](#_ENREF_71),[72](#_ENREF_72),[73](#_ENREF_73),[78](#_ENREF_78),[82](#_ENREF_82),[84](#_ENREF_84)], four on Asian physicians [[15](#_ENREF_15),[53](#_ENREF_53),[69](#_ENREF_69),[83](#_ENREF_83)], two on Australian physicians [[40](#_ENREF_40),[52](#_ENREF_52)], one on South American physicians [[50](#_ENREF_50)], and one reported African physicians [[56](#_ENREF_56)].

**Periods (epoch) of time***:* Statistics on *SMR for suicides among physicians* were computed among a period from 1-5 years in six studies [[7](#_ENREF_7),[58](#_ENREF_58),[63](#_ENREF_63),[65](#_ENREF_65),[66](#_ENREF_66),[68](#_ENREF_68)], a period from 6-10 years in seven studies [[8](#_ENREF_8),[54](#_ENREF_54),[56](#_ENREF_56),[61](#_ENREF_61),[62](#_ENREF_62),[67](#_ENREF_67),[82](#_ENREF_82)], from 11-15 years in four studies [[46](#_ENREF_46),[55](#_ENREF_55),[57](#_ENREF_57),[70](#_ENREF_70)], from 16-20 years in three studies [[41](#_ENREF_41),[59](#_ENREF_59),[69](#_ENREF_69)], from 21-25 years in one study [[60](#_ENREF_60)], from 36-40 years in two studies [[52](#_ENREF_52),[64](#_ENREF_64)], from 46-50 years in one study [[71](#_ENREF_71)], and from 71-80 years in one study [[72](#_ENREF_72)]. Statistics on *percentages of suicide by specialties* were computed among a period from 1-5 years in one study [[16](#_ENREF_16)], from 6-10 years in two studies [[47](#_ENREF_47),[51](#_ENREF_51)], from 11-15 years in three studies [[15](#_ENREF_15),[40](#_ENREF_40),[83](#_ENREF_83)], from 16-20 years in one study [[45](#_ENREF_45)], and was not mentioned in one study [[43](#_ENREF_43)]. Statistics on *the number of physicians dead by suicide among all deaths in physicians,* were computed among 1-5 years in four studies [[16](#_ENREF_16),[39](#_ENREF_39),[42](#_ENREF_42),[44](#_ENREF_44)], for 6-10 years in two studies [[50](#_ENREF_50),[51](#_ENREF_51)], from 11-15 years in one study [[46](#_ENREF_46)], from 16-20 years in three studies [[41](#_ENREF_41),[49](#_ENREF_49),[53](#_ENREF_53)], from 21-25 years in one study [[48](#_ENREF_48)], and from 36-40 years in one study [[52](#_ENREF_52)]. Statistics on *the number of deaths by suicide in physicians among all deaths by suicide in the general population*, were computed among a period from 1-5 years in three studies [[8](#_ENREF_8),[36](#_ENREF_36),[37](#_ENREF_37)], from 11-16 years in two studies [[15](#_ENREF_15),[82](#_ENREF_82)], from 20-25 years in one study [[1](#_ENREF_1)], from 26-30 years in one study [[5](#_ENREF_5)], and from 35-40 years in two studies [[34](#_ENREF_34),[35](#_ENREF_35)]. Statistics on *the number of physicians having done suicide attempt among all the physicians* were computed among a period from 1-5 years in three studies [[73](#_ENREF_73),[77](#_ENREF_77),[85](#_ENREF_85)], and 6-10 years in two studies [[47](#_ENREF_47),[75](#_ENREF_75)]. Statistics on *the number of physicians with suicidal ideation among all the physicians* were computed among a period from 1-5 years in five studies [[74](#_ENREF_74),[75](#_ENREF_75),[76](#_ENREF_76),[77](#_ENREF_77),[85](#_ENREF_85)], from 6-10 years in two studies [[75](#_ENREF_75),[84](#_ENREF_84)], and for 16-20 years in one study [[78](#_ENREF_78)].

**Types of specialties***:* Eight studies reported the number of physicians died by suicide by specialties [[15](#_ENREF_15),[16](#_ENREF_16),[40](#_ENREF_40),[43](#_ENREF_43),[45](#_ENREF_45),[47](#_ENREF_47),[51](#_ENREF_51),[83](#_ENREF_83)]. Eighteen specialties were described: ten medical specialties, seven surgical specialties, and the general practitioners. Medical specialties were internal medicine in six studies [[15](#_ENREF_15),[16](#_ENREF_16),[43](#_ENREF_43),[47](#_ENREF_47),[51](#_ENREF_51),[83](#_ENREF_83)], psychiatrists in six studies [[15](#_ENREF_15),[16](#_ENREF_16),[40](#_ENREF_40),[43](#_ENREF_43),[45](#_ENREF_45),[47](#_ENREF_47)], anesthesiologists in six studies [[15](#_ENREF_15),[16](#_ENREF_16),[40](#_ENREF_40),[43](#_ENREF_43),[47](#_ENREF_47),[83](#_ENREF_83)], radiologists in five studies [[15](#_ENREF_15),[16](#_ENREF_16),[43](#_ENREF_43),[45](#_ENREF_45),[51](#_ENREF_51)], pediatricians in five studies [[15](#_ENREF_15),[16](#_ENREF_16),[43](#_ENREF_43),[51](#_ENREF_51),[83](#_ENREF_83)], pathologists in two studies [[16](#_ENREF_16),[43](#_ENREF_43)], cardiologists in one study [[83](#_ENREF_83)], neurologists in one study [[83](#_ENREF_83)], emergency in one study [[83](#_ENREF_83)], and dermatologists in two study [[15](#_ENREF_15),[83](#_ENREF_83)]. Surgical specialties were general surgeons in four studies [[15](#_ENREF_15),[16](#_ENREF_16),[43](#_ENREF_43),[51](#_ENREF_51)], obstetricians in five studies [[15](#_ENREF_15),[16](#_ENREF_16),[43](#_ENREF_43),[47](#_ENREF_47),[83](#_ENREF_83)], orthopedists in two studies [[15](#_ENREF_15),[16](#_ENREF_16)], ophtalmologists in three studies [[15](#_ENREF_15),[16](#_ENREF_16),[83](#_ENREF_83)], ears, nose and throat in one study [[15](#_ENREF_15)], urologists in one study [[83](#_ENREF_83)], and plastic surgeons in the last one [[15](#_ENREF_15)]. General practitioners were described in six studies [[16](#_ENREF_16),[40](#_ENREF_40),[43](#_ENREF_43),[45](#_ENREF_45),[47](#_ENREF_47),[51](#_ENREF_51)].

**Outcomes and aims of the studies**

The principal aim of most included studies was to compare suicide rates between physicians and general population [[5](#_ENREF_5),[7](#_ENREF_7),[8](#_ENREF_8),[34](#_ENREF_34),[54](#_ENREF_54),[55](#_ENREF_55),[56](#_ENREF_56),[57](#_ENREF_57),[58](#_ENREF_58),[59](#_ENREF_59),[60](#_ENREF_60),[61](#_ENREF_61),[62](#_ENREF_62),[63](#_ENREF_63),[64](#_ENREF_64),[65](#_ENREF_65),[66](#_ENREF_66),[67](#_ENREF_67),[68](#_ENREF_68),[69](#_ENREF_69),[70](#_ENREF_70),[71](#_ENREF_71),[72](#_ENREF_72),[82](#_ENREF_82),[83](#_ENREF_83),[84](#_ENREF_84),[85](#_ENREF_85)]. The aim of seven studies was to examine causes of mortality among physicians [[41](#_ENREF_41),[42](#_ENREF_42),[48](#_ENREF_48),[50](#_ENREF_50),[51](#_ENREF_51),[52](#_ENREF_52),[53](#_ENREF_53)], and to compare these causes with those of the general population in five studies [[35](#_ENREF_35),[44](#_ENREF_44),[46](#_ENREF_46),[49](#_ENREF_49),[88](#_ENREF_88)]. In addition, the principal aim of seven studies was to examine the characteristics of physicians’ suicides [[15](#_ENREF_15),[16](#_ENREF_16),[40](#_ENREF_40),[43](#_ENREF_43),[45](#_ENREF_45),[47](#_ENREF_47),[62](#_ENREF_62)], and for three studies, it was to compare physicians and non-physicians risk factors of suicide [[1](#_ENREF_1),[36](#_ENREF_36),[38](#_ENREF_38)]. Moreover, the principal aim of two studies was to examine correlates between depression and suicide attempts among physicians [[73](#_ENREF_73),[85](#_ENREF_85)], the correlate between suicidal ideation and suicide attempts among physicians in two studies [[75](#_ENREF_75),[85](#_ENREF_85)], to compare suicidal ideation between physicians and general population for one study [[77](#_ENREF_77)], and to identify risk factors of suicidal ideation in physicians for four studies [[74](#_ENREF_74),[76](#_ENREF_76),[78](#_ENREF_78),[84](#_ENREF_84)]. Finally, the aim was not mentioned for one study [[37](#_ENREF_37)].

**Study designs**

Included studies were mainly retrospective. Seven studies were cross-sectional with questionnaires send on suicidal ideation and suicide attempts [[57](#_ENREF_57),[74](#_ENREF_74),[75](#_ENREF_75),[76](#_ENREF_76),[77](#_ENREF_77),[84](#_ENREF_84),[85](#_ENREF_85)]. Only one study was a historical prospective cohort study [[59](#_ENREF_59)].
