# Supplementary figures and images for: Suicide among physicians and health-care workers: A systematic review and meta-analysis
Source: PLoS One. 2019 Dec 12;14(12):e0226361. doi: 10.1371/journal.pone.0226361 (PMC6907772; doi:10.1371/journal.pone.0226361)

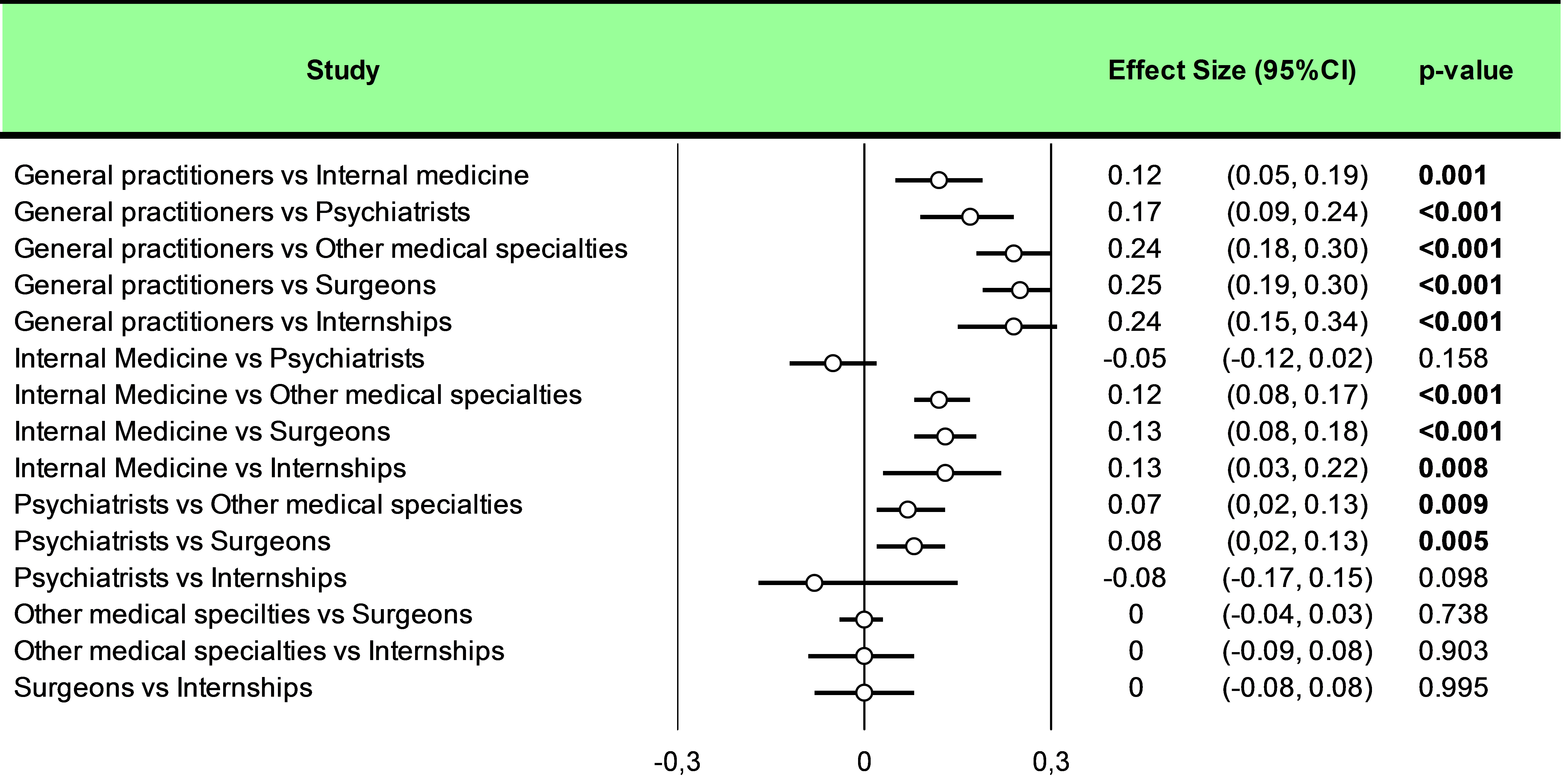

Supplement: S1 Fig — (TIF) [file pone.0226361.s003.tif]

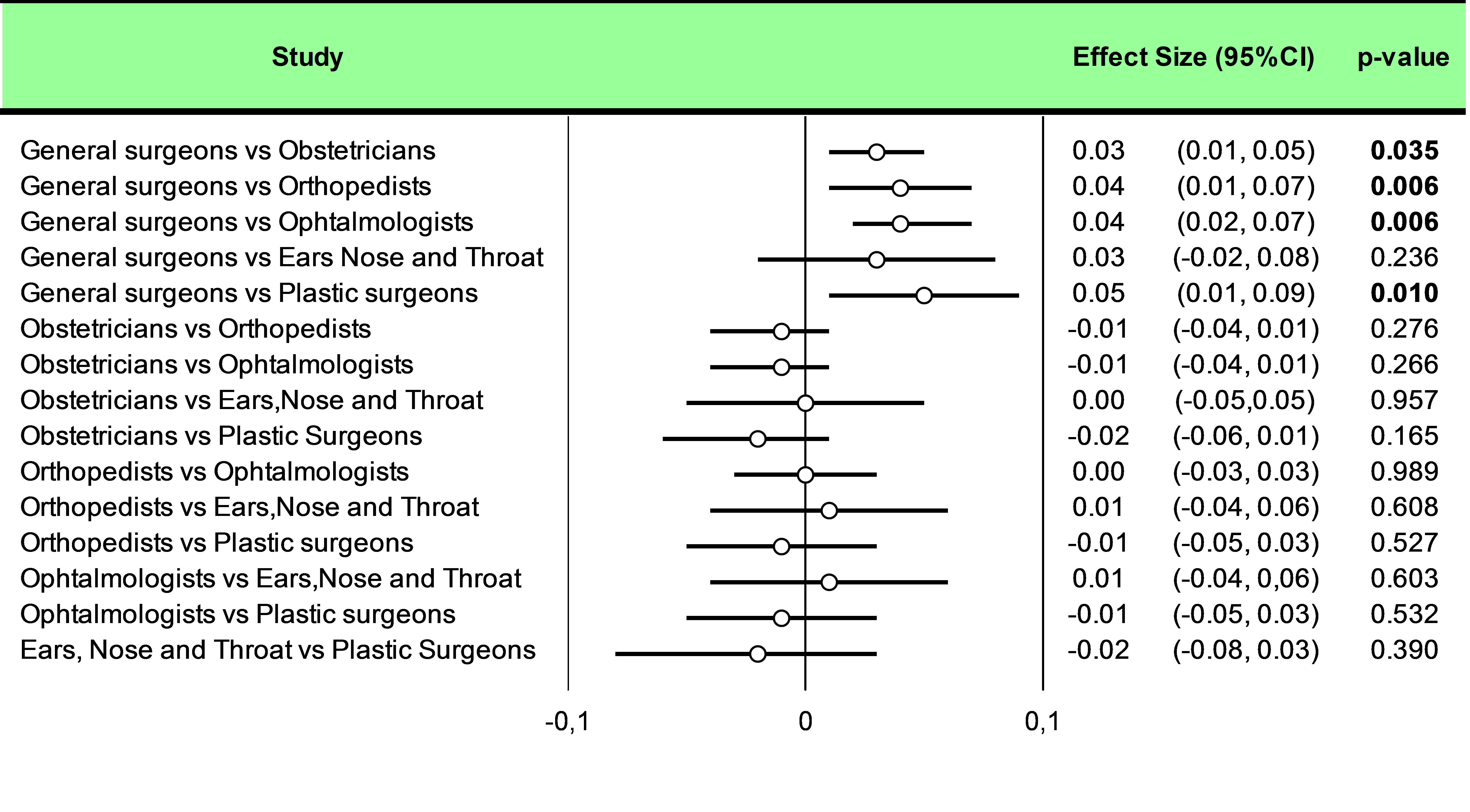

Supplement: S2 Fig — (TIF) [file pone.0226361.s004.tif]

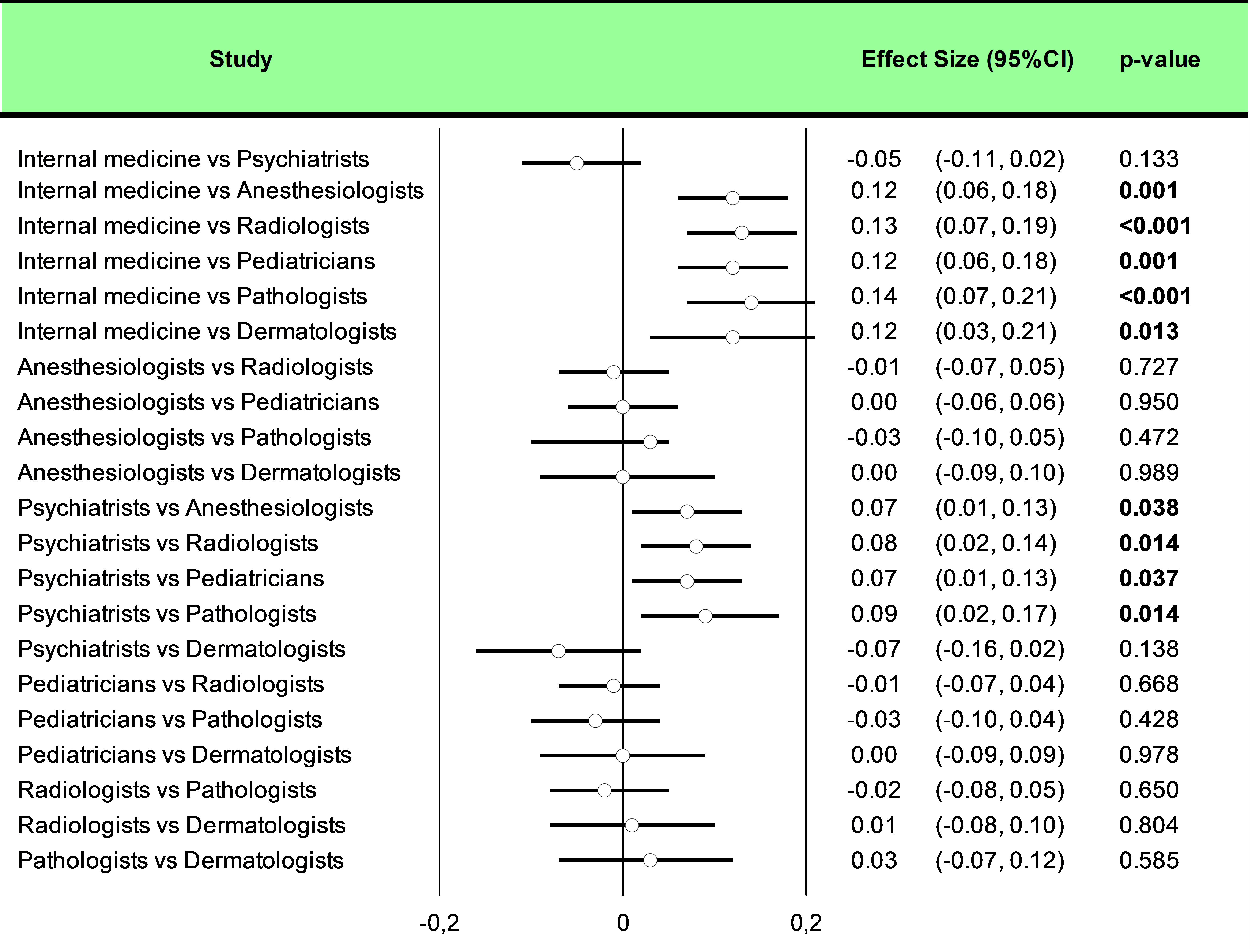

Supplement: S3 Fig — (TIF) [file pone.0226361.s005.tif]

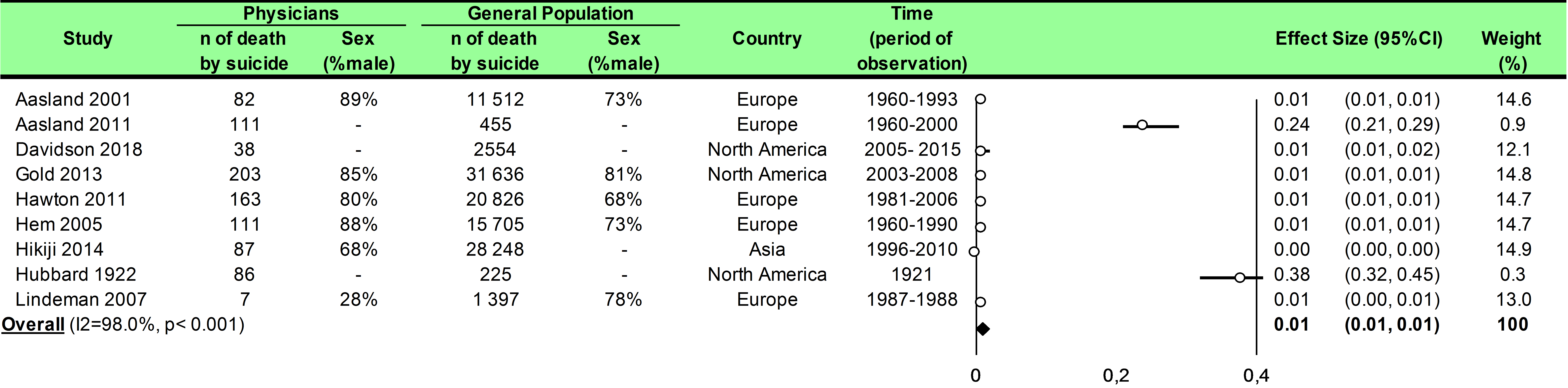

Supplement: S4 Fig — (TIF) [file pone.0226361.s006.tif]

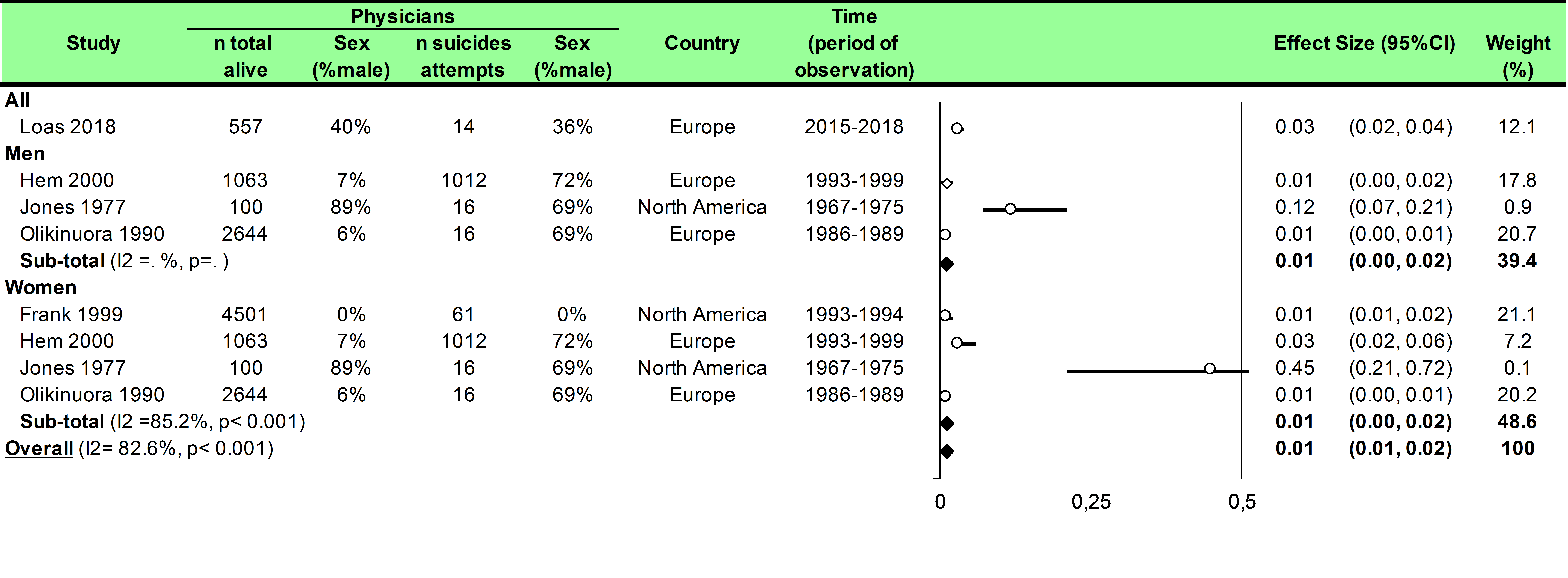

Supplement: S5 Fig — (TIF) [file pone.0226361.s007.tif]
