# Supplementary material for: Suicide among physicians and health-care workers: A systematic review and meta-analysis
Source: PLoS One. 2019 Dec 12;14(12):e0226361. doi: 10.1371/journal.pone.0226361 (PMC6907772; doi:10.1371/journal.pone.0226361)
Supplement: Attachment — Submitted filename: 2019-11-12_PlosOne_ResponseToReviewers.docx [file pone.0226361.s009.docx]

*Psychological & Psychosocial Stress*

– National Centre for Scientific Research, **CNRS**, UMR 6024

– **UniversitY ClERMONT AUVERGNE** (**UCA**)

63 000 Clermont-Ferrand, France

[Frederic.Dutheil@uca.fr](mailto:Frederic.Dutheil@uca.fr)

*Preventive & Occupational Medicine*

– **UniversitY HOSPITAL OF CLERMONT-FERRAND (CHU)**

63000 Clermont-Ferrand

France; tel: +33(0)6 88 22 48 48

[fdutheil@chu-clermontferrand.fr](mailto:fdutheil@chu-clermontferrand.fr)

*Faculty of Health,*

*School of Exercise Science*
 – **Australian Catholic University** (**ACU**)

Melbourne, VIC 3065

Australia

[Frederic.Dutheil@acu.edu.au](mailto:Frederic.Dutheil@acu.edu.au)

Frédéric Dutheil, MD, PhD, HDR

*Professor in Medicine*, Occupational and Environmental Health, University Hospital of Clermont-Ferrand (CHU)

*Head of the ‘Psychological & Psychosocial Stress’ team*, CNRS, University Clermont Auvergne (UCA)

*Clinical fellow of the Australian Catholic University* (ACU)

November 12^th^, 2019

Joerg Heber

Editor-in-Chief

PlosOne

Dear Editor,

My coauthors and I welcomed the review of our Manuscript PONE-D-19-21600 entitled **“Suicide among physicians and health-care workers A systematic review and meta-analysis”**. We have addressed the comments of the reviewers in a revised manuscript and enclose a point-by-point response.

**Review Comments to the Author**

Thank you for the opportunity to review the revised manuscript, “Suicide among physicians and health-care workers: A systematic review and meta-analysis.” I thank the authors for their thoughtful and thorough response to reviewers. I believe the manuscript is much improved. Only two minor points remain.

[REPLY] Thank you for your positive comment.

- The authors added the sentence “For example, recent years saw tentative to regulate working time of physicians, such as in Europe with its European Working Time Directive (EWTD).” I believe there may be a word missing following tentative.

[REPLY] Thank you for your comment. The sentence now reads: “For example, there were tentative to regulate working time of physicians over the recent years, such as in Europe with its European Working Time Directive (EWTD).”

- Thank you for the added explanation regarding the exclusion of students. However, what about the relevance to interns (some fields consider interns as students, while others do not)? Are they expected to have responsibilities that do not reflect traditional students? Were they included in the previous meta-analysis?

[REPLY] Thank you for your comment. We included internship students in our meta-analysis because previous meta-analyses did not include interns (medical students included were year 1 to 5 or 6 in all meta-analyses on prevalence of suicids or suicidal ideations – Puthran et al. 2016 Rotenstein et al. 2016 and Zeng et al. 2019) and because they could have similar responsibilities to senior practitioners. We added the following sentence within the Methods section: “Students were excluded because of the difference in responsibilities in comparisons with health-care workers, and because of the existence of previous recent meta-analyses focusing specifically on health-care students [21,22,23,24]; we included interns because they were not included in the aforementioned meta-analyses on prevalence of suicides, suicide attempts or suicidal ideation, and because they could have similar responsibilities to senior practitioners.”

- Thank you for clarifying the inclusion criteria regarding information on healthcare workers vs. the general population in the methods section. However, did this influence the studies included? That is, were studies that did not information on the general population excluded from the review? If so, this would suggest it may be necessary to re-review the excluded studies.

[REPLY] Thank you for your comment. It did not influence the studies included as data in the general population were not mandatory (it was needed only for some meta-analysis for between groups comparison: health care workers versus general population). We added the following sentence within the Methods section: “When data were available, we also collected data from a control group (such as general population) for comparisons purposes.”

We hope our work will be considered favorably and look forward to hearing from you.

Sincerely yours,

Frédéric Dutheil
